# From neuroinflammation to immune reprogramming: Nanozyme-Integrated nanoplatforms for neuroimmune modulation in Parkinson's disease

**DOI:** 10.1016/j.mtbio.2026.103569

**Published:** 2026-08-17

**Authors:** Shu Zhu, Zhongting Wang, Xiaoxi Shi, Shanshan Zhong, Ghulam Md Ashraf, Kailei Fu, Yugang Li

**Affiliations:** a Department of Rehabilitation, Shengjing Hospital of China Medical University, Shenyang, China; b Department of Neurology/Stroke Center, The First Hospital of China Medical University, Liaoning Provincial Key Laboratory of Big Data for Neurological Diseases, Shenyang, China; c Department of Vascular Surgery/Thyroid Surgery, The First Hospital of China Medical University, Shenyang, Liaoning, China; d Department of Biomedical Sciences, College of Medicine, Gulf Medical University, Ajman, United Arab Emirates

**Keywords:** Parkinson's disease, Neuron-immune crosstalk, Neuroinflammation, Microglia, Astrocytes, α-Synuclein, Nanomedicine, Neuroprotection, Disease modification, Neural repair

## Abstract

Parkinson's disease (PD) is a progressive neuroimmune disorder in which dysregulated neuron–glia–immune crosstalk drives chronic neuroinflammation, α-synuclein pathology, blood–brain barrier dysfunction, and mitochondrial failure, establishing interconnected neuroimmune pathways as therapeutic targets. Aberrant neuron–immune crosstalk promotes chronic neuroinflammation, oxidative stress, mitochondrial dysfunction, blood-brain barrier (BBB) disruption, and α-synuclein pathology, thereby accelerating neurodegeneration. Understanding these interconnected mechanisms has identified multiple neuroimmune pathways as promising therapeutic targets. This review summarizes the cellular and molecular basis of physiological and pathological neuron-immune communication in PD, highlighting the roles of microglial activation, astrocyte reactivity, adaptive immune responses, inflammatory signaling networks, and neurovascular dysfunction. We further discuss emerging neuroimmune-targeted interventions aimed at restoring immune homeostasis and slowing disease progression. Particular emphasis is placed on nanozyme-integrated nanoplatforms that function simultaneously as nanozymatic catalysts and immunomodulatory adjuvants, including enzyme-mimetic nanozymes (SOD/CAT/GPx-like), single-atom catalysts, and nanozyme-integrated nanoplatforms. These platforms not only scavenge reactive oxygen species, degrade α-synuclein aggregates, and reinforce BBB integrity through localized catalytic reactions, but also reprogram innate and adaptive immune responses by modulating microglial polarization toward neuroprotective phenotypes, suppressing pro-inflammatory cytokine cascades (TNF-α, IL-1β, IL-6), and supporting regulatory T cell (Treg) responses. We examine how these nanozyme-integrated nanoplatforms can be combined with lipid-based nanoparticles, polymeric carriers, biomimetic systems, and extracellular vesicles (EVs) to achieve synergistic targeted drug delivery, gene modulation, neuroprotection, and neural repair. Finally, we address the translational challenges and prospects for merging neuroimmunology, nanocatalysis, and adjuvant engineering in PD therapy.

## Highlights

• Parkinson's disease is increasingly recognized as a neuroimmune disorder driven by dysregulated neuron-immune crosstalk.
• α-Synuclein aggregation, microglial activation, astrocyte reactivity, and BBB dysfunction form an interconnected pathogenic network.
• Neuroimmune pathways, including inflammatory signaling and adaptive immune responses, are promising therapeutic targets.
• Advanced nanoplatforms enable targeted blood-brain barrier crossing, immunomodulation, gene delivery, and α-synuclein clearance in the treatment of Parkinson's disease.
• Multifunctional nanomedicines offer new opportunities for neuroprotection, neural repair, and disease modification in patients with Parkinson's disease.

## Graphical abstract

Nanoplatform-based therapies target dysregulated neuroimmune crosstalk in Parkinson's disease to promote immunomodulation, α-synuclein clearance, neuroprotection, restoration of the blood-brain barrier, and neural repair, ultimately supporting disease modification and functional recovery.

**Figure:**
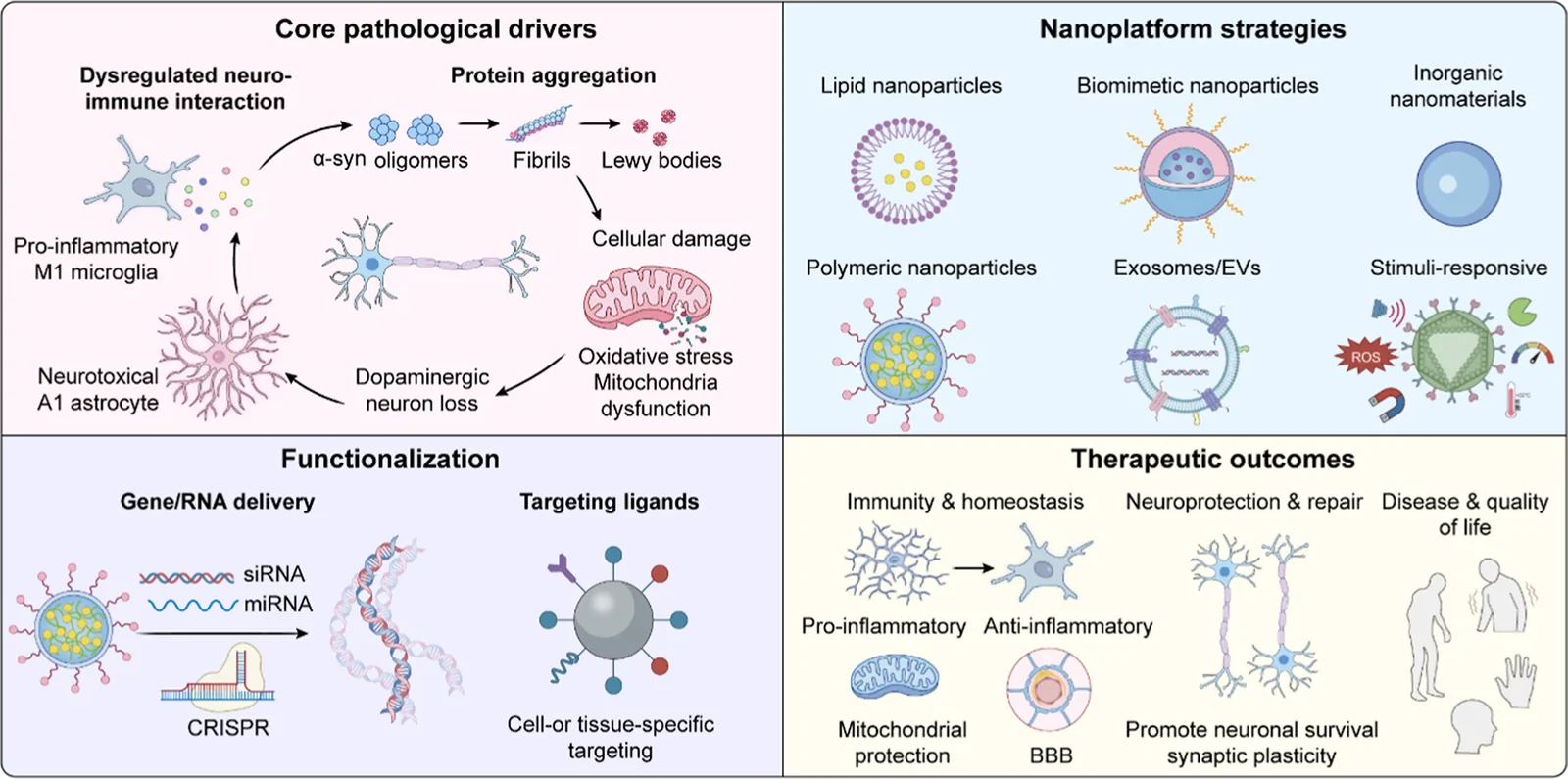

## 1 Introduction

### 1.1 Global burden and clinical challenges of PD

Parkinson's disease is the second most prevalent neurodegenerative disorder after Alzheimer's disease and has emerged as a global public health challenge. There has been a significant increase in the prevalence of PD over the last 30 years, mainly due to better diagnostic awareness, population aging, and increased environmental exposure [1,2]. Currently, it is estimated that over 10 million people worldwide have PD, and the number of people with PD will continue to grow significantly in the next few decades. Clinically, PD is characterized by a motor syndrome with resting tremor, bradykinesia, rigidity, postural instability, and a wide range of non-motor symptoms, including cognitive impairment, depression, sleep disturbances, autonomic dysfunction, and sensory abnormalities. These symptoms collectively contribute to progressive functional decline, reduced quality of life, and a substantial socioeconomic burden on patients, as reported by Meer et al. (2024) and Stroe and Pretorian (2024) [3,4].

While significant advances have been made in the symptomatic treatment of PD, such as levodopa replacement therapy, dopamine agonists, and deep brain stimulation, the focus of current treatments is on symptomatic management rather than on disease mechanisms. Consequently, even optimally treated patients continue to experience neurodegeneration. The absence of effective disease-modifying drugs underscores the need to understand PD pathogenesis more thoroughly and determine therapies that can help delay, stop, or reverse the disease progression [5,6].

### 1.2 Beyond dopamine loss: PD as a neuroimmune disorder

PD has been primarily considered a movement disorder secondary to progressive degeneration of the substantia nigra pars compacta (SNpc) and a decrease in dopamine levels in the striatum. Lewy bodies are another distinguishing pathological feature, mainly composed of α-synuclein protein aggregates that accumulate within neurons. While these features are central to disease pathogenesis, it has become increasingly clear that PD is much more than selective neuron loss and is marked by profound abnormalities in neuroimmune regulation [7,8].

Under physiological conditions, communication between neurons and resident immune and glial cells, including microglia, astrocytes, and regulatory T cells (Tregs), is dynamic and tightly regulated [9,10]. Key soluble mediators governing these interactions include brain-derived neurotrophic factor (BDNF), glial cell line-derived neurotrophic factor (GDNF), interleukin-10 (IL-10), and transforming growth factor-β (TGF-β), which collectively maintain CNS homeostasis, preserve neuronal integrity, and calibrate immune responses. It has recently been discovered that both the innate and adaptive immune systems are extensively involved in PD pathogenesis. Neuroinflammatory responses have been observed in post-mortem brain tissues, experimental models, and clinical samples of patients with PD. Disease progression is heavily influenced by activated microglia, reactive astrocytes, increased inflammatory cytokine levels, infiltrating peripheral immune cells, and disturbances in immune signaling pathways. Additionally, several genes associated with PD involved in immune regulation, lysosomal function, mitochondrial homeostasis, and inflammatory responses have been identified, providing further evidence that immune dysfunction plays a major role in PD [11,12].

**Fig. 1:**
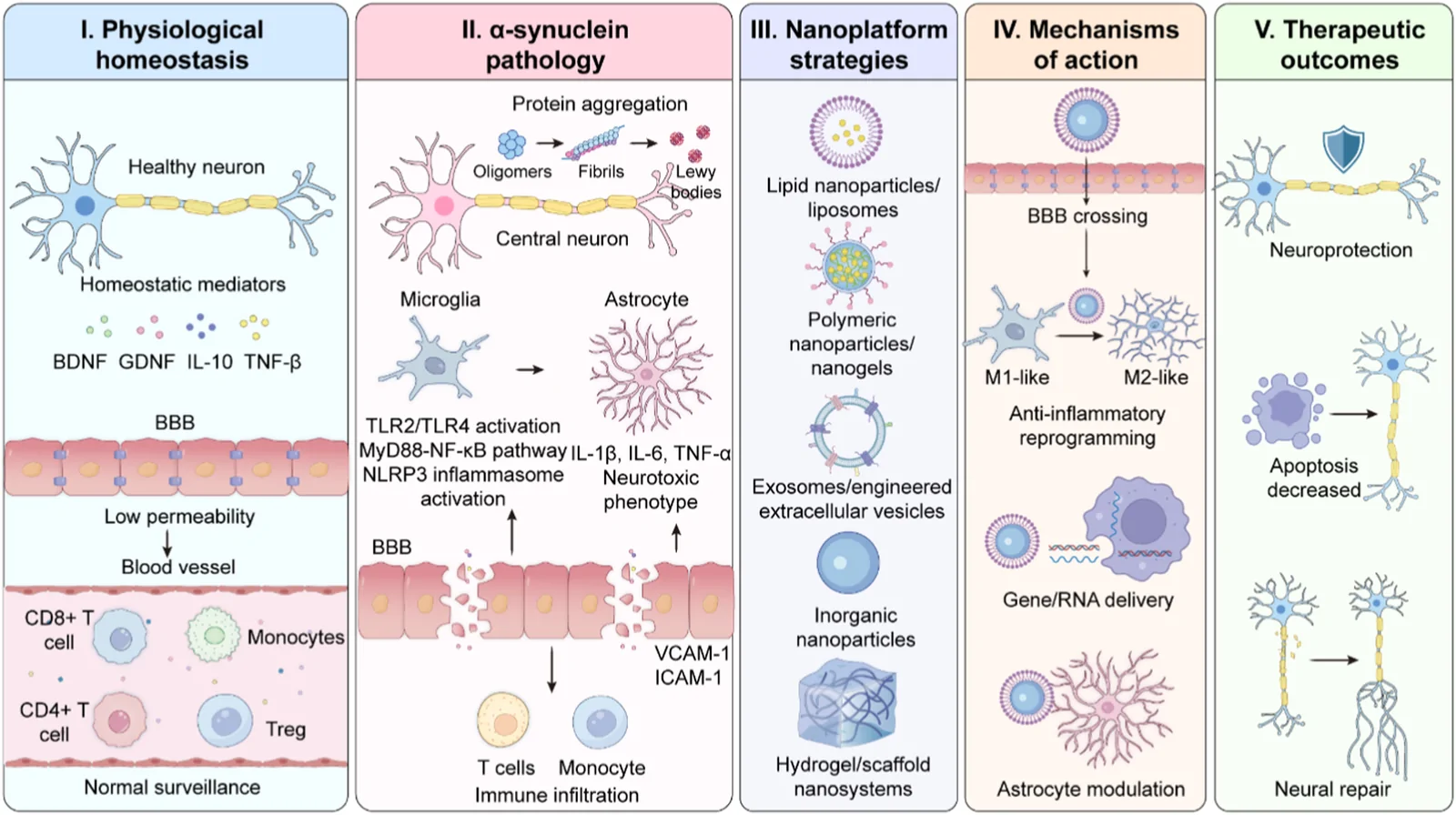
Overview of neuroimmune crosstalk, pathogenesis of PD, and nanoplatform-based therapeutic strategies. Physiological neuron–glia–immune interactions maintain CNS homeostasis, whereas α-synuclein aggregation drives microglial and astrocytic activation, BBB dysfunction, peripheral immune infiltration, and chronic neuroinflammation. Emerging nanoplatforms offer targeted approaches for BBB crossing, α-synuclein clearance, immune modulation, neuroprotection, disease modification, and neural repair [13]. Created by the authors using Adobe Illustrator.

These observations have led to a paradigm shift and a deeper understanding of PD as a neuroimmune disease characterized by chronic inflammation between the immune and nervous systems. Understanding these mechanisms is essential for elucidating disease progression and identifying novel therapeutic targets for neuronal dysfunction and chronic inflammation (Fig. 1) [14].

### 1.3 Emerging importance of neuron-immune interactions in disease progression

The CNS is traditionally considered immune-privileged and protected from peripheral immune influence. Recent research has shown that bidirectional communication occurs between neurons and various immune cell types under normal and disease conditions. In a healthy brain, neuron-immune interactions lead to the coordinated regulation of signaling between neurons, microglia, astrocytes, and peripheral immune regulators, resulting in synaptic remodeling, tissue maintenance, neuroprotection, and homeostatic regulation, as demonstrated by Müller and Benedetto (2025) [13].

These interactions are dysregulated in PD, and oligomers of misfolded α-synuclein eventually convert into fibrils and progressively accumulate as Lewy body aggregates. These pathological species are responsible for neuronal injury, which consists of synaptic dysfunction, mitochondrial impairment, oxidative stress, and neuronal death. At the same time, extracellular α-synuclein is also a potent danger-associated molecular pattern that may activate microglial cells via innate immune receptors, such as Toll-like receptor 2 (TLR2) and Toll-like receptor 4 (TLR4). These pathways activate inflammatory signaling pathways, such as the NF-κB and NLRP3 inflammasome pathways, leading to the production of pro-inflammatory mediators, such as IL-1β, IL-6, and TNF-α [15,16].

Additional inflammatory signals from microglia contribute to the conversion of astrocytes to a more reactive state, diminishing many of their neuroprotective functions and leading to neuronal injury. In this context, neuronal injury, gliosis, and inflammatory responses are linked and feed into each other in a self-perpetuating cycle that leads to neurodegeneration [17,18].

Peripheral immune mechanisms and resident glial cell populations play important roles in disease progression. BBB dysfunction facilitates the infiltration of T lymphocytes, monocytes, and macrophages into the CNS, further increasing BBB permeability. These infiltrating immune cells engage with resident microglia and astrocytes to drive neuroinflammatory responses and promote neuronal injury. New evidence has shown that systemic inflammation, peripheral immune dysregulation, and gut–brain communication play roles in the initiation and propagation of α-synuclein pathology. Taken together, these results suggest that neuron-immune crosstalk is a key mechanism that connects protein aggregation, neuroinflammation, BBB disruption, and progressive neurodegeneration in patients with PD, as shown by Gullotta et al. (2023) and Zhang et al. (2022) [19,20].

### 1.4 Nanomedicine as a transformative approach for PD

Many molecular targets have been identified; however, some biological and pharmacological challenges have restricted the successful therapeutic interventions in PD. The BBB is one of the greatest challenges in delivering therapeutic agents to the affected brain regions. Traditional medications may have suboptimal BBB penetration, be rapidly metabolized in systemic circulation, have poor bioavailability, poorly target specific diseases, and have undesirable off-target effects that reduce their clinical utility [21,22].

Nanomedicine is a promising approach to overcoming these limitations. Nanoplatforms can be designed to increase drug stability, BBB penetration, circulation time, and targeted delivery to specific cell populations. Various nanosystems, including lipid nanoparticles (LNPs), liposomes, polymeric nanoparticles, nanogels, inorganic nanomaterials, biomimetic cell membrane-coated nanoparticles, EVs, and hydrogel-based nanosystems, have demonstrated remarkable therapeutic potential in preclinical PD models [22,23].

Importantly, nanotechnology offers opportunities beyond conventional drug delivery. Advanced nanoplatforms can be engineered to cross the BBB, promote α-synuclein clearance, modulate microglial and astrocytic functions, deliver genes and nucleic acids, reduce oxidative stress, restore mitochondrial function, support neurotrophic function, and stimulate regenerative processes in the brain. These multifunctional properties offer the promise of nanomedicine for treating multiple pathological processes associated with PD. These strategies may contribute to achieving neuroprotection, disease modification, and neural repair, which are important therapeutic targets for next-generation PD treatment strategies (Fig. 1) [24,25].

### 1.5 Scope of the work

The first section of this review discusses the cellular and molecular mechanisms underlying neuron-immune interactions in the normal and pathological brain, with a particular focus on the mechanisms involved in neuroimmune dysfunction in PD. We then discuss ongoing therapeutic approaches targeting neuroimmune pathways and provide a detailed account of the emerging nanotechnology-based platforms that facilitate neural repair, modify diseases, and promote neuroprotection. Finally, we discuss the challenges, clinical implications, and future directions of precision nanomedicine strategies for changing the treatment landscape of PD. Fig. 1 presents the overall conceptual framework that connects physiological neuron-immune homeostasis, α-synuclein-mediated neuroimmune dysfunction, BBB disruption, peripheral immune involvement, and opportunities for novel therapies using nanoplatforms. Several recent reviews have examined the neuroinflammatory pathogenesis of PD and nanoplatform-based drug delivery strategies for PD in isolation. The present review distinguishes itself by: (1) integrating biocatalytic nanozyme activity with immunomodulatory adjuvant function as a unified dual-mechanism therapeutic concept; (2) systematically mapping nanoplatform design principles onto the specific neuroimmune dysfunction network of PD, from α-synuclein-triggered microglial activation to BBB breakdown and peripheral immune infiltration; and (3) providing a comprehensive treatment of catalytic-adjuvant nanoplatforms spanning innate immune reprogramming, adaptive immune modulation, antioxidant nanocatalysis, gene regulation, and neural repair within a single mechanistic framework.

### 1.6 Literature search strategy

A systematic literature search was conducted using the PubMed/MEDLINE, Web of Science, Scopus, and Google Scholar databases. The primary search period spanned January 2015 to May 2026, with key foundational studies published before 2015 included were essential for mechanistic context. Search terms were used in Boolean combinations and included: Parkinson's disease, neuroinflammation, neuroimmune, neuron-immune crosstalk, α-synuclein, microglia, astrocytes, oligodendrocytes, blood-brain barrier, nanomedicine, nanoparticles, nanoplatform, nanozyme, nanocatalysis, biocatalysis, lipid nanoparticles, exosomes, extracellular vesicles, neuroprotection, disease modification, neural repair, and gene delivery. Articles were screened based on the title, abstract, and full-text relevance to the review's scope. Duplicate records and retracted studies were excluded from the review. The reference lists of the included articles were hand-searched to identify relevant studies that were not captured by the database search.

## 2 Cellular and molecular basis of neuron-immune crosstalk in PD

### 2.1 Neuroanatomical landscape of the nigrostriatal system

The nigrostriatal pathway is the primary neural pathway impaired in PD and the primary anatomical framework in which neuroimmune interactions occur. This pathway originates from dopaminergic neurons in the SNpc. It broadly innervates the dorsal striatum, which plays an important role in motor coordination, reward processing, learning, and cognition, all of which are controlled by dopamine release. El-Mansoury et al. (2023) reported that the degeneration of dopaminergic neurons is a key pathological finding in PD. The nigrostriatal system is a highly integrated neuroimmune microenvironment involving several different types of neural, glial, vascular, and immune cells [26].

**Fig. 2:**
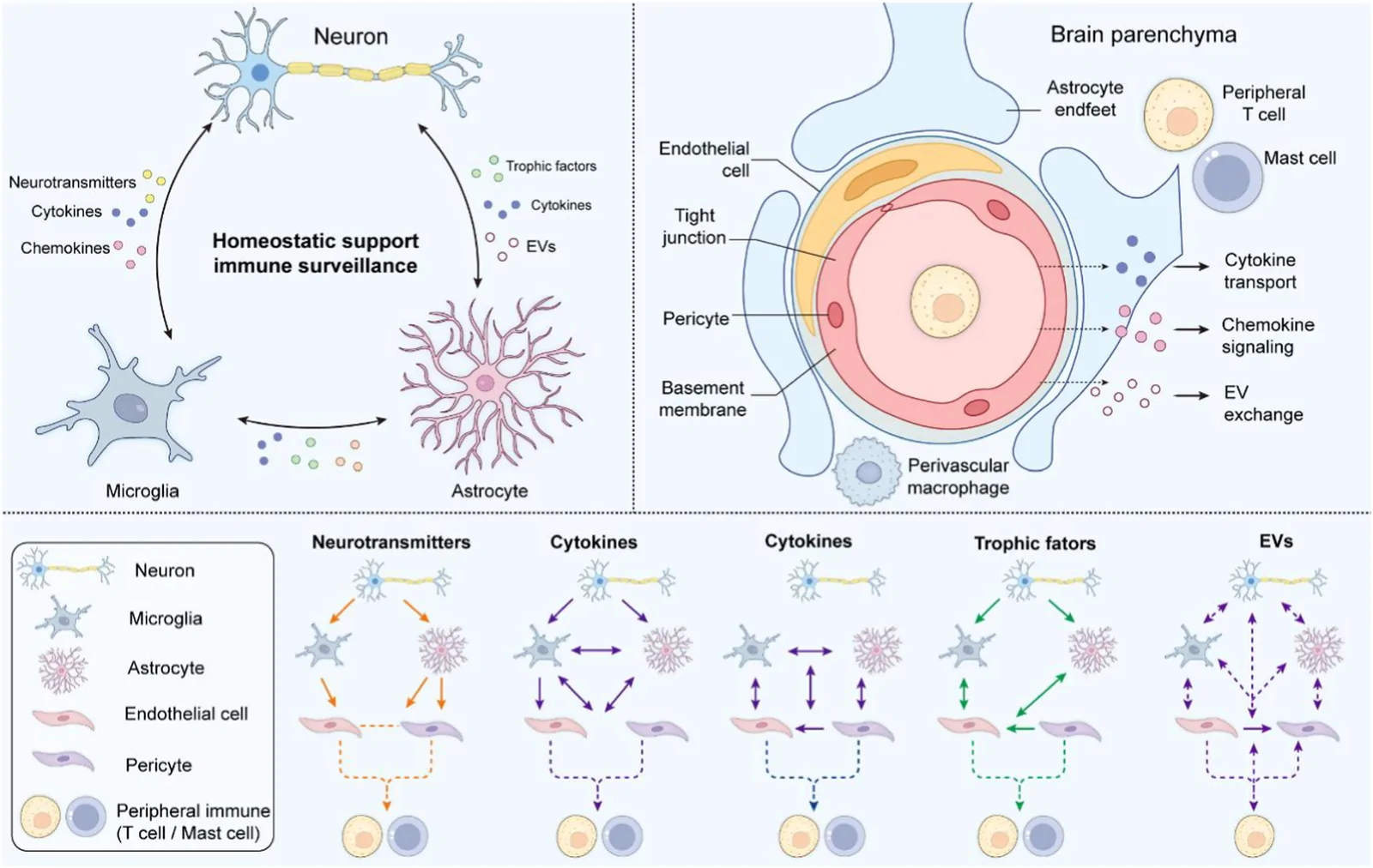
Cellular architecture and molecular basis of physiological neuron-immune communication in the healthy brain. This model was adapted and synthesized from current concepts of brain cell communication and neuroimmune regulation. Neurons, microglia, astrocytes, endothelial cells, pericytes, and peripheral immune cells interact through neurotransmitters, cytokines, chemokines, trophic factors, and extracellular vesicles (EVs) to maintain central nervous system homeostasis and immune surveillance. Adapted from Saavedra et al. [27] and Kovacs et al. [28]. (a) Homeostatic communication among neurons, microglia, and astrocytes in the healthy brain. Bidirectional signaling mediated by neurotransmitters, cytokines, chemokines, trophic factors, and EVs coordinates neuronal activity, immune surveillance, synaptic maintenance, and neuroprotective support, thereby preserving the brain's physiological functions [27,28]. (b) Neurovascular communication at the blood–brain barrier (BBB). Endothelial cells, pericytes, astrocytic end-feet, and perivascular macrophages form a specialized neurovascular unit that regulates the controlled exchange of cytokines, chemokines, and EVs between circulating immune cells and the brain parenchyma while maintaining barrier integrity and immune homeostasis [28]. (c) Principal signaling networks governing physiological neuron–glia–vascular–immune interactions. Neurotransmitters, cytokines, chemokines, trophic factors, and EVs establish multidirectional communication among neurons, microglia, astrocytes, endothelial cells, pericytes, and peripheral immune cells, supporting tissue maintenance, neurovascular function, and coordinated immune regulation in the healthy brain. Adapted from Saavedra et al. (Frontiers in Cell and Developmental Biology, CC BY 4.0) and Kovacs et al. (Nature Reviews Immunology, adapted with permission from Springer Nature, permission reference [27,28].

The nigrostriatal system comprises not only neurons but also microglia, astrocytes, oligodendrocytes, endothelial cells, pericytes, perivascular macrophages, and peripheral immune cells, all of which support the maintenance of CNS homeostasis. *In vivo*, the regulation of these cell populations is mediated by EVs, neurotransmitters, cytokines, chemokines, and trophic factors, creating a network of interconnected interactions that play roles in immune surveillance, synaptic plasticity, metabolic regulation, and tissue maintenance (Fig. 2). Table 1 shows the main neural, glial, vascular, and immune cell populations, their major signaling molecules, and their homeostatic functions in physiological neuron-immune communication [49,50].

**Table 1:** Neural, glial, vascular, and immune cell populations are involved in physiological neuron-immune crosstalk.

Cell Type	Primary Function	Major Signaling Molecules	Role in Homeostasis	References
Dopaminergic neurons	Neurotransmission and neural circuit regulation	Dopamine, ATP, CX3CL1 (fractalkine)	Regulation of glial activity and maintenance of neuronal signaling	[29,30]
Microglia	Immune surveillance and phagocytosis	TGF-β, BDNF, IL-10, cytokines	Synaptic maintenance, debris clearance, and immune monitoring	[31,32]
Astrocytes	Metabolic support and neurotransmitter regulation	Glutamate transporters, cytokines, and trophic factors	BBB support, neurotransmitter recycling, and neuronal protection	[33,34]
Oligodendrocytes	Axonal insulation and metabolic support	Myelin-associated proteins, growth factors	Maintenance of axonal integrity and efficient signal conduction	[35,36]
Endothelial cells	Vascular regulation and barrier function	Adhesion molecules, nitric oxide, and cytokines	BBB maintenance and regulation of molecular transport	[37,38]
Pericytes	Neurovascular stabilization	PDGF-BB, TGF-β, angiogenic factors	BBB integrity and vascular homeostasis	[39,40]
Perivascular macrophages	Perivascular immune surveillance	Cytokines, chemokines, and scavenger receptors	Immune monitoring at the neurovascular interface	[41,42]
Tregs	Immune tolerance	IL-10, TGF-β	Suppression of excessive inflammation and maintenance of immune balance	[43,44]
Monocytes/Macrophages	Peripheral immune surveillance and repair	Cytokines, growth factors, chemokines	Tissue maintenance, repair, and CNS–peripheral immune communication	[45,46]
Mast cells and parenchymal T cells	Neuroimmune signaling and immune surveillance	Histamine, cytokines, chemokines	Regulation of local inflammatory responses and immune homeostasis	[47,48]

The susceptibility of dopaminergic neurons is partly due to their extensive arborization, high energy demands, and sensitivity to oxidative stress. Thus, impaired communication between various components of the homeostatic network, such as between neurons, glia, vessels, and immune cells, can have dramatic consequences on neuron survival and make the nigrostriatal system especially vulnerable to neurodegenerative processes [51,52].

### 2.2 Physiological communication between neurons and immune cells

Neuron-immune communication is a tightly regulated process that is integral to normal CNS function. Neurons, glial cells, blood vessels, and peripheral immune cells are not independent entities but constantly communicate with each other to coordinate tissue maintenance, immune surveillance, and neuroprotection in the brain, as reported by Godbout (2024) and Khalid et al. (2025) [53,54].

Neurons actively regulate immune cell activity via membrane-bound ligands, soluble mediators, neurotransmitters, trophic factors, and EVs. Dopamine, glutamate, γ-aminobutyric acid (GABA), acetylcholine, and ATP are neurotransmitters that regulate the activation of microglia and astrocytes and affect cytokine production. Neuronal activity can directly influence inflammatory responses through direct action on microglia and astrocytes via dopamine receptors. In contrast, ATP released during neuronal signaling is an important mediator of microglial surveillance and process extension [50,55].

Cytokines and chemokines also play a role in communication among neurons, glial cells, vascular cells, and peripheral immune compartments. Neurons and glial cells secrete neurotrophic factors that promote cell survival, maintain synapses, and repair tissues. In addition, EVs are vital mediators of intercellular communication, as they carry proteins, lipids, messenger RNAs, microRNAs, and other bioactive molecules from neighboring cells to distant cells. Neurons also produce inhibitory molecules that inhibit over-activation of the immune system. The most relevant are CD200 and CX3CL1 (fractalkine) and their respective receptors on microglial cells, which are involved in immune quiescence. These various types of communication allow the neurons to modulate immune function constantly, and at the same time are supported and nourished by the other surrounding glial elements [56,57].

### 2.3 Microglial surveillance and homeostatic functions

In the healthy brain, microglia are resident innate immune cells that act as major regulators of immune surveillance. Homeostatic microglia are highly dynamic, with constant extension and retraction of cellular processes that sense the extracellular environment and respond rapidly to changes in tissue integrity [58,59].

In addition to their classical immune functions, microglia play a crucial role in the development and maintenance of the CNS. They are involved in synaptic pruning, controlling neurogenesis, removing apoptotic cells and cell debris by phagocytosis, and synaptic remodeling in adulthood. Microglia release trophic molecules such as brain-derived neurotrophic factor (BDNF) that promote the survival, plasticity, and adaptive function of neurons [60,61].

Neuron–astrocyte communication is essential for maintaining microglial homeostasis. Microglial phenotype and responsiveness are regulated by cytokines, chemokines, and trophic factors derived from astrocytes, whereas neuronal signals, such as fractalkine and CD200, are involved in regulating excessive inflammatory activity. Emerging evidence suggests that communication between cells via EVs is a mechanism of microglial regulation by transferring signaling molecules between cells. These mechanisms allow microglia to assimilate environmental signals and respond appropriately and homeostatically [62,63]. Recent single-cell RNA sequencing studies have revealed that microglia are not a homogeneous population but comprise multiple transcriptomically distinct subtypes with specific spatiotemporal distributions and functional states in the PD brain. Disease-associated microglia (DAM) are characterized by downregulation of homeostatic genes (CX3CR1, P2RY12) and upregulation of phagocytic and lipid metabolism genes (TREM2, ApoE, LPL), enabling enhanced α-synuclein clearance but also heightened inflammatory potential. Microglial neurodegenerative phenotype (MGnD) cells, driven by TREM2 signaling, share features with DAM but exhibit greater lipid droplet accumulation and impaired phagocytic function. Interferon (IFN)-responsive microglia, characterized by upregulation of IFITM3 and MHC class II genes, are prominent in the substantia nigra of PD patients and may amplify T-cell-mediated neuroinflammation. This cellular heterogeneity has important implications for nanomedicine: nanoplatforms targeting surface receptors preferentially expressed on DAM (e.g., TREM2 ligands) or exploiting homeostatic microglial endocytosis pathways may achieve substantially different therapeutic outcomes depending on the predominant microglial state in the target brain region.

### 2.4 Astrocyte–neuron-immune interactions

Astrocytes are the most prevalent glial cells in the CNS and play an essential role in maintaining CNS homeostasis. They play roles in regulating neurotransmitter recycling, ion balance, energy metabolism, synaptic activity, and BBB integrity. Extensive cellular interactions and the presence of specialized vascular end-feet enable astrocytes to directly communicate with neurons, endothelial cells, pericytes, and immune cells, placing them at the center of neuroimmune and neurovascular homeostasis [33,64].

Gorbacheva et al. (2019) and Klegeris (2021) reported that astrocytes actively participate in immune system regulation by producing cytokines, chemokines, trophic factors, and EVs. These signals help create anti-inflammatory conditions and facilitate neuronal survival under physiological conditions. The glutamate uptake capacity of astrocytes prevents excitotoxicity, whereas the release of neurotrophic molecules is linked to the maintenance and functional stability of neurons [65,66].

Astrocytes and microglia play vital roles in maintaining CNS homeostasis through bidirectional communication. The activation of astrocyte and microglial gene expression, as well as functional states, is regulated by signals from each other, and the regulation of microglial activation involves the release of cytokines from astrocytes, metabolic support, and EV-mediated signaling by astrocytes. These interactions are coordinated to regulate immune responses and aid tissue repair after physiological stress or minor injury [63,67]. Astrocytes, like microglia, exhibit significant heterogeneity in their reactive states under neuroinflammatory conditions. The classical binary A1/A2 framework, in which A1 astrocytes are neurotoxic (induced by IL-1α, TNF-α, and complement C1q from activated microglia) and A2 astrocytes are neuroprotective (promoted by ischemic stimuli), has provided a useful conceptual scaffold, but emerging single-cell transcriptomic evidence demonstrates a spectrum of disease-stage-dependent reactive states beyond this binary classification. In PD, A1-like neurotoxic astrocytes predominate in the substantia nigra and exhibit reduced glutamate transporter expression, impaired α-synuclein clearance, and enhanced secretion of complement proteins and pro-inflammatory lipid mediators. Notably, A1 and A2 astrocytes differ substantially in their uptake capacity for nanoparticles and their responsiveness to nanoparticle-delivered immunomodulatory cargo, which should be considered when designing astrocyte-targeting nanoplatforms for PD.

### 2.5 Peripheral immune contributions to CNS homeostasis

The CNS was once considered an immune-privileged organ, but it is now known that peripheral immune cells play important roles in brain homeostasis. In normal physiology, immune surveillance is maintained by regulated interactions between blood cells in the immune system and CNS-associated structures, such as the meninges, choroid plexus, and perivascular space of the CNS [68,69].

The neurovascular unit, composed of endothelial cells, pericytes, astrocytic end-feet, basement membrane components, and perivascular macrophages, represents an important connection between the peripheral immune system and the CNS. It is a highly specialized organization that controls the exchange of molecules and cells between the circulation and brain parenchyma while maintaining BBB integrity. These mechanisms, such as cytokine transport, chemokine-mediated signaling, and extracellular vesicle exchange, allow communication between peripheral immune cells and CNS-resident cells without affecting barrier function (Fig. 2) [70,71].

Antigen presentation, cytokine production, and interaction with resident glial populations are all ways in which peripheral immune cells affect CNS physiology. Monocytes and macrophages are involved in tissue surveillance and repair, whereas Tregs are involved in immune tolerance and help to limit excessive inflammatory responses. Under physiological conditions, local immune regulation may occur by other immune cells, such as parenchymal T cells and mast cells [72,73].

The lymphatic drainage pathways and the newly described meningeal lymphatic network also promote interactions between the CNS and the peripheral immune system. The gut–brain axis plays an important role in regulating communication between the neuroimmune system and gut metabolites and/or immune responses, as reported by Jiang and Sun (2024) and Park et al. (2025) [74,75].

### 2.6 Molecular signaling pathways governing neuroimmune communication

The integrated network of neuron-immune crosstalk signaling pathways controls cellular activation, survival, communication, and inflammation. Five main groups of mediators mediate physiological communication within the CNS: trophic factors, cytokines, chemokines, neurotransmitters, and EVs. All these mediators work together to coordinate interactions between neurons, microglia, astrocytes, endothelial cells, pericytes, and peripheral immune cells and maintain tissue homeostasis and immune balance (Fig. 2) [76,77].

The CX3CL1/CX3CR1 and CD200/CD200R systems are two of the most significant regulatory pathways involved in regulating inhibitory communication between neurons and microglia and helping maintain microglial quiescence. Purinergic signaling mediated by ATP and P2 receptors controls microglial migration, surveillance, and sensory responsiveness. TGF-β signaling also promotes immune regulation and tissue repair by supporting anti-inflammatory phenotypes and maintaining cellular homeostasis [78,79].

Other pathways involve the activation of NF-κB, JAK/STAT, and MAPK pathways by cytokines, which control inflammatory genes and cellular adaptation. Chemokine signaling regulates the positioning and migration of cells in neural tissues, whereas trophic factors promote neuronal survival and maintain synaptic integrity and tissue. EVs offer an additional level of regulation, as they enable the exchange of proteins, lipids, and nucleic acids between cells, allowing multidirectional communication between neural, glial, vascular, and immune compartments [80,81].

These pathways are interconnected and form an extremely coordinated network of signals that maintain CNS integrity under normal physiological conditions. The relative contributions of these pathways determine whether neuroimmune interactions maintain homeostasis or drive pathological inflammation and neurodegeneration.

## 3 Neuroimmune dysregulation in PD pathogenesis

Dysregulation of the neuroimmune system is a major pathway in PD progression. It is more than just a result of neuronal damage. The loss of dopaminergic neurons and α-synuclein accumulation are the two most prominent pathological features of PD. However, recent studies suggest that chronic interactions between neurons, glial cells, peripheral immune cells, and inflammatory mediators create a chronic inflammatory environment that drives disease progression. Protein aggregation, mitochondrial dysfunction, oxidative stress, BBB dysfunction, and genetic susceptibility contribute to the development of self-propagating cycles of neuronal injury and neurodegeneration, known as neuroimmune dysfunction [82,83].

### 3.1 α-Synuclein aggregation as a trigger of neuroimmune responses

α-Synuclein is a presynaptic protein that plays a role in synaptic vesicle trafficking, neurotransmitter release, and the maintenance of neuronal homeostasis. Under physiological conditions, it is mainly present in the soluble conformation. In contrast, genetic mutations, post-translational modifications, environmental factors, and protein clearance defects during aging facilitate misfolding and aggregation. Soluble α-synuclein oligomers are precursors of fibrils and Lewy body-like inclusions, which are pathological hallmarks of PD [84,85].

In addition to their direct neurotoxicity, α-synuclein aggregates act as danger-associated molecular patterns (DAMPs) that trigger innate immune responses. Extraneuronal α-synuclein may interact with pattern recognition receptors, such as TLR2, TLR4, RAGE, and scavenger receptors, which are present in microglia and astrocytes. Activation of these receptors promotes inflammatory signaling pathways, especially NF-κB, leading to the secretion of proinflammatory cytokines and chemokines [15,86].

Importantly, α-synuclein pathology has prion-like properties. The misfolded species spread to the interconnected regions of the brain via EVs, endocytic uptake, and direct cell-to-cell transfers. Microglia and astrocytes take up α-synuclein; however, as the amount of α-synuclein increases, clearance mechanisms, including autophagy and lysosomes, become overwhelmed, and these cells become a source of chronic inflammation [87]. Thus, α-synuclein is not only a pathological protein but also a significant cause of neuroimmune deregulation in PD. One of the most important pathogenic cascades, as shown by Fan et al. (2006) and He et al. (2021), that leads to neuronal dysfunction and neuroimmune activation is the progressive conversion of soluble α-synuclein into toxic oligomers, fibrils, and Lewy body-like inclusions (Fig. 4) [88,89].

### 3.2 Microglial activation and sustained neuroinflammation

Microglial activation is among the earliest detectable neuroimmune alterations in prodromal PD, reportedly preceding substantial dopaminergic neuron loss in the substantia nigra pars compacta, an observation supported by TSPO-PET neuroimaging studies in LRRK2 mutation carriers and REM-sleep behavior disorder cohorts, and by post-mortem analyses of Braak stage I–III tissue. There is a consistent pattern of elevated glial activation in neuroimaging studies, postmortem examinations, and experimental models of PD-affected brain regions, including the substantia nigra, striatum, and other brain regions. Transient activation plays a role in tissue repair and pathogen defense, whereas chronic activation plays a role in neurodegeneration [90,91].

Upon recognition of aggregated α-synuclein by microglial TLR2 and TLR4, downstream signaling cascades trigger NF-κB nuclear translocation, NLRP3 inflammasome assembly, excessive oxidative stress, and autophagy dysfunction, ultimately driving the transcription and secretion of pro-inflammatory mediators, including TNF-α, IL-1β, IL-6, nitric oxide, and ROS, that disrupt neuronal function and accelerate dopaminergic neurodegeneration (Fig. 4) [92,93].

One feature of PD-associated neuroinflammation is the development of a positive feedback loop. The disruption of neurons leads to the release of additional α-synuclein aggregates, ATP, and mitochondrial components, all of which are DAMPs and therefore further activate microglia. Activated microglia amplify the neuronal stress response through a persistent inflammatory response, leading to chronic neuroinflammation, even in the absence of external stress. This self-sustaining positive feedback loop, fueled by α-synuclein aggregates, ATP, and mitochondrial damage-associated molecular patterns (DAMPs) released from stressed neurons, can sustain chronic neuroinflammation for months to years even after the initial exogenous trigger is eliminated. Recent studies have indicated that there are populations of microglia with varying activation states and that therapeutic interventions may be developed that induce a shift in microglial activation from neurotoxic to protective [[94], [95], [96]]. Beyond these autonomous microglial responses, astrocytes serve as critical second-order amplifiers of the neuroinflammatory cascade, their reactive transformation directly shaped by, and in turn sustaining, the microglial activation environment described above.

### 3.3 Astrocyte reactivity and neurotoxic transformation

Astrocytes play a crucial role in maintaining neuronal homeostasis by recycling neurotransmitters, providing metabolic support, regulating ion balance, and preserving the integrity of the BBB. However, astrocytes undergo reactive changes in PD, including changes in gene expression, functional reprogramming, and morphological alterations [97,98].

Within damaged brain regions, there is an accumulation of reactive astrocytes, which exhibit increased expression of inflammatory mediators and stress-related pathways. These signals, such as activated microglia-derived IL-1α, TNF-α, and complement component C1q, induce the expression of a neurotoxic phenotype in astrocytes, characterized by the loss of many neuroprotective properties. Simultaneously, extracellular α-synuclein activates TLR4-dependent signaling, the JNK/p38 pathway, oxidative stress responses, endoplasmic reticulum stress, and lysosomal dysfunction [99,100].

**Fig. 3:**
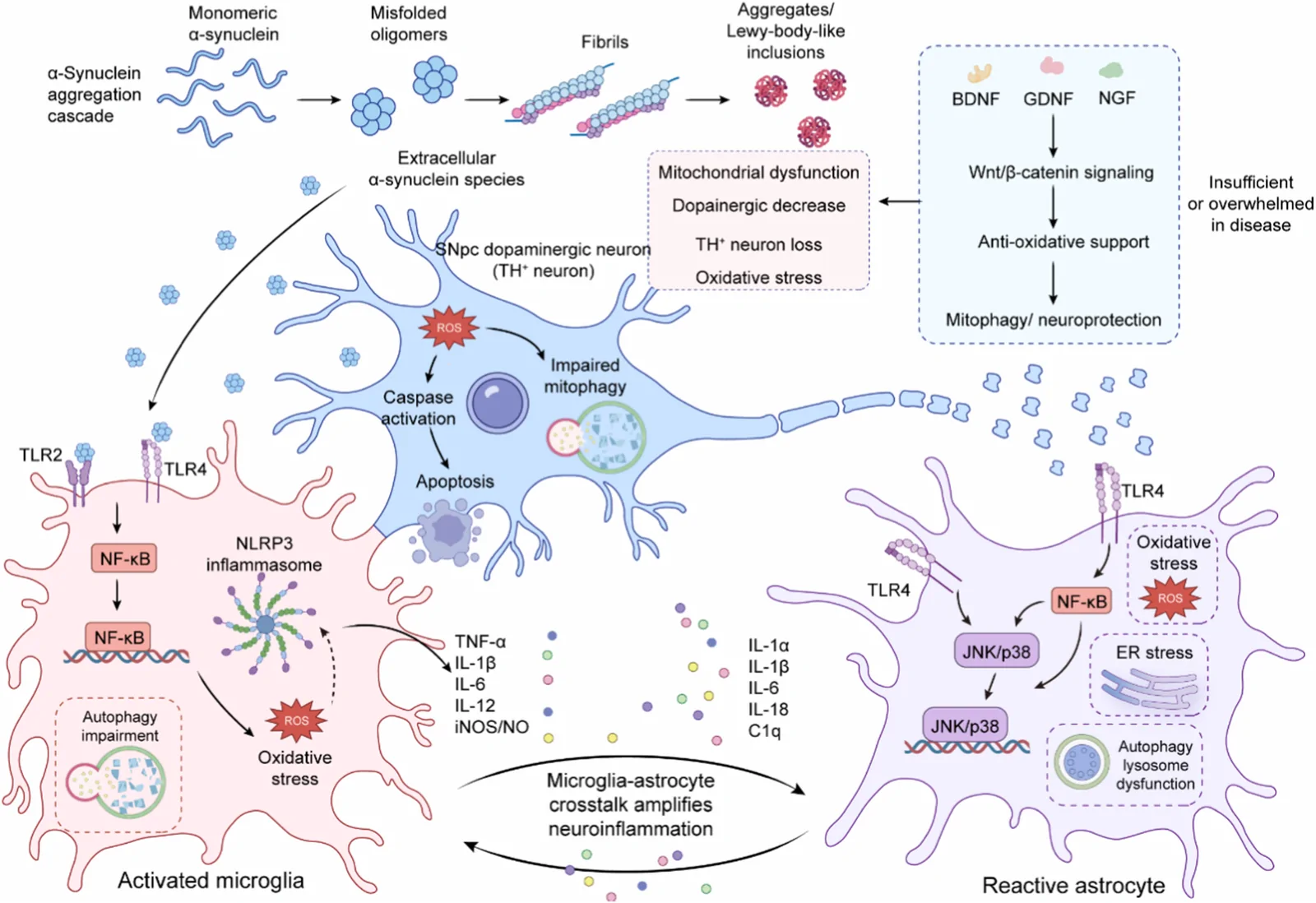
α-Synuclein-driven neuroimmune dysregulation in PD. Misfolded α-synuclein activates microglia and astrocytes through TLR-dependent signaling pathways, promoting NF-κB activation, NLRP3 inflammasome assembly, oxidative stress, impaired autophagy and mitophagy, inflammatory cytokine release, and progressive dopaminergic neurodegeneration [103]. Adapted with permission.

**Fig. 4:**
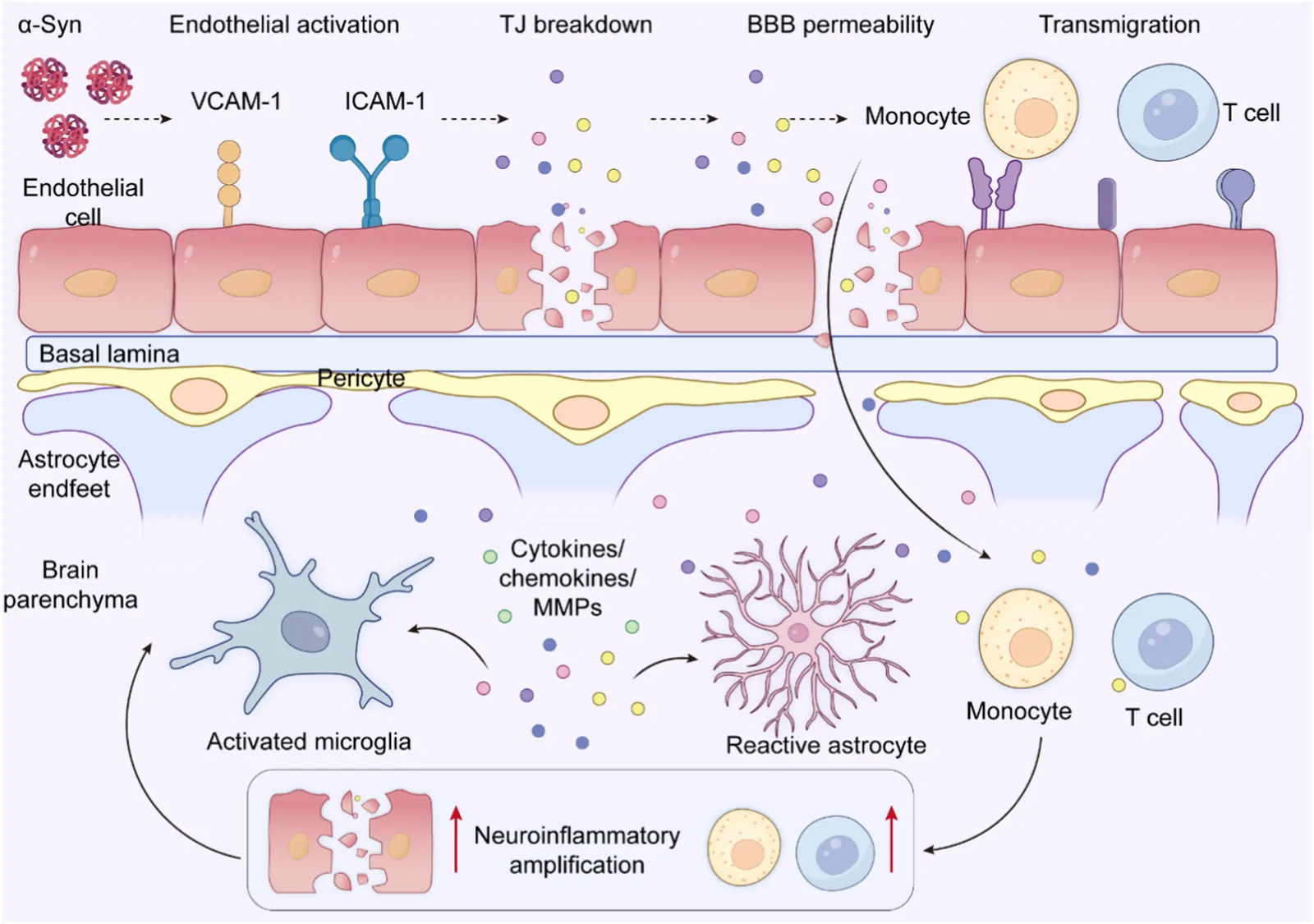
BBB dysfunction and peripheral immune infiltration in patients with PD. α-Synuclein-induced endothelial activation promotes VCAM-1 and ICAM-1 expression, tight-junction disruption, increased BBB permeability, and recruitment of monocytes and T cells into the CNS, thereby amplifying neuroinflammatory responses and neuronal injury [104]. Adapted with permission.

While astrocytes initially help to clear α-synuclein, they alter degradative pathways when excessive amounts accumulate, resulting in the release of ROS, nitric oxide, IL-1β, IL-6, and other inflammatory mediators. Astrocytic dysfunction also results in decreased glutamate uptake and increased extracellular glutamate levels, leading to excitotoxic neuronal injury [101,102]. In bidirectional interactions with activated microglia, reactive astrocytes serve as key amplifiers of chronic neuroinflammation and dopaminergic degeneration (Fig. 3). Activated microglial cells and reactive astrocytes create a feed-forward loop of inflammation that perpetuates oxidative stress, dysregulates proteostatic mechanisms, and promotes neuronal injury (Fig. 4) [94,105].

### 3.4 Peripheral immune cell recruitment and adaptive immunity

There is growing evidence that the peripheral immune system plays an important role in the pathogenesis of PD. Changes in circulating immune cell subsets and increased inflammatory markers and antigen-specific immune function have been observed throughout the disease stages [106,107].

The adaptive immune system is significant in disease progression. CD4^+^ and CD8^+^ T cells have been found in the substantia nigra and surrounding brain areas of patients with PD. These infiltrating cells may recognize α-synuclein-derived peptides presented by major histocompatibility complex (MHC) class I and class II molecules on microglia and other antigen-presenting cells [108,109].

CD8^+^ T cells directly induce neuronal apoptosis, whereas CD4^+^ helper T cells stimulate inflammation by secreting cytokines. Both Th1 and Th17 cell subsets produce elevated levels of IFN-γ, IL-17, and TNF-α, thereby further amplifying microglial activation and neuronal damage. In contrast, Tregs regulate the immune system and ensure tolerance to excessive inflammation. Thus, there is a decrease in Treg activity in PD, which may be responsible for maintaining chronic neuroimmune activation and disease progression [110,111].

### 3.5 Cytokines, chemokines, and inflammatory networks

Inflammatory molecules mediate molecular crosstalk between neurons, glial cells, and peripheral immune cells. In patients with PD, serum, CSF, and brain tissue contain increased cytokine and chemokine levels [112].

The cytokines most consistently elevated are tumor necrosis factor-alpha (TNF-α), interleukin-1β (IL-1β), Interleukin-6 (IL-6), and interferon-gamma (IFN-γ). These mediators induce inflammatory signaling pathways, promote oxidative stress, disrupt neuronal metabolism, and promote BBB dysfunction. CCL2, CXCL10, and CXCL12 are other chemokines that are important for amplifying inflammation by attracting monocytes, macrophages, and T-cells to inflammatory sites [113,114].

Importantly, inflammatory networks act as a network, and the production of cytokines induces the production of chemokines, which in turn leads to the infiltration of more immune cells that produce more inflammatory mediators. This orchestrated amplification leads to a broad inflammatory response in the brain, involving multiple cell types and regions. Table 2 shows the key inflammatory mediators, signaling pathways, and immune-associated genes previously identified to be associated with PD [138,139].

**Table 2:** Key inflammatory mediators, signaling pathways, and immune-associated genes involved in dysregulation of the neuroimmune system in PD.

Category	Representative Components	Functional Significance in PD	Refences
Pro-inflammatory cytokines	TNF-α, IL-1β, IL-6, IFN-γ	It promotes neuroinflammation, neuronal injury, oxidative stress, and glial activation.	[115,116]
Inflammasome cytokines	IL-1β, IL-18	Mediate NLRP3 inflammasome-dependent inflammatory responses.	[117,118]
Anti-inflammatory cytokines	IL-10, TGF-β	Suppresses excessive inflammation and supports immune homeostasis.	[20,119]
Chemokines	CCL2, CXCL10, CXCL12	Recruit monocytes, macrophages, and T cells to inflamed tissues	[120,121]
Innate immune receptors	TLR2, TLR4, RAGE, P2X7	Recognition of α-synuclein aggregates and danger-associated signals	[122,123]
Inflammasome machinery	NLRP3, ASC, caspase-1	Amplification of inflammatory signaling and cytokine maturation	[124,125]
Major signaling pathways	NF-κB, JAK/STAT, MAPK	Regulation of inflammatory gene expression and immune activation	[126,127]
Stress-response pathways	JNK, p38 MAPK, Wnt/β-catenin	Regulation of cellular stress responses, inflammation, and neuroprotection	[128,129]
Oxidative stress mediators	ROS, nitric oxide, NADPH oxidase	Promote oxidative damage and inflammatory amplification	[130,131]
Adaptive immune factors	MHC-II, CD4^+^ T cells, CD8^+^ T cells, Tregs	Antigen presentation, immune regulation, and neurodegeneration	[132,133]
PD-associated immune genes	SNCA, LRRK2, GBA1	Regulation of α-synuclein biology, lysosomal function, and inflammatory responses	[134,135]
Mitochondrial quality-control genes	PINK1, PRKN, DJ-1	Regulation of mitophagy, oxidative stress, and immune activation	[136,137]

### 3.6 BBB dysfunction and neuroimmune amplification

The BBB is a specialized neurovascular interface comprising endothelial cells, pericytes, astrocytic end-feet, and extracellular matrix (ECM) elements that control molecular and cellular exchanges between the circulation and CNS. BBB integrity must be maintained to preserve neural homeostasis and prevent inappropriate immune activation, as shown by Sharif et al. (2018) and Zhang (2023) [140,141].

Evidence suggests that BBB dysfunction is a hallmark of PD. α-Synuclein activation of endothelial cells leads to the increased expression of adhesion molecules, such as VCAM-1 and ICAM-1. Inflammatory cytokines, chemokines, and matrix metalloproteinases promote the disruption of tight junctions and enhance vascular permeability. These changes allow the passage of circulating cytokines, antibodies, monocytes, and lymphocytes into the CNS [142,143].

Infiltrating immune cells interact with activated microglia and reactive astrocytes, further potentiating inflammatory responses and promoting early neuronal injury. In parallel, inflammatory mediators released by the CNS continue to compromise BBB components, creating a vicious cycle of BBB dysfunction and neuroimmune amplification. These processes have the potential to affect the delivery of therapeutic agents and response to treatment (Fig. 4) and can be associated with disease progression [19,144].

### 3.7 Mitochondrial dysfunction, oxidative stress, and inflammasome activation

Mitochondrial dysfunction is a crucial component of PD pathogenesis. Moradi Vastegani et al. (2023) reported that the energy requirement of dopaminergic neurons is extremely high, and these neurons are highly susceptible to defects in mitochondrial respiration, dynamics, and quality control [145].

Impaired mitophagy and mitochondrial dysfunction result in excessive ROS production, oxidative stress, and activation of apoptotic pathways. ROS cause damage to proteins, lipids, and nucleic acids and facilitate the aggregation of α-synuclein. Furthermore, oxidative stress activates redox-sensitive signaling pathways, including NF-κB, which directly connects metabolic dysfunction to neuroimmune activation [146,147].

The NLRP3 inflammasome, activated downstream of mitochondrial ROS, mitochondrial DNA release, and lysosomal rupture (see Section 3.2 for the full TLR–NF-κB–NLRP3 pathway), drives caspase-1-mediated IL-1β and IL-18 maturation, sustaining chronic neuroinflammation and progressive dopaminergic loss (Fig. 3) [148,149]. Accordingly, mitochondrial dysfunction, oxidative stress, and NLRP3 signaling have collectively emerged as disease-modifying targets.

### 3.8 Genetic determinants of neuroimmune dysregulation

Genetic research has yielded valuable clues regarding the molecular pathways connecting neurodegeneration and immune dysfunction in patients with PD. Many PD-associated genes affect inflammation, mitochondrial metabolism, protein degradation, and lysosomal function [150,151].

Mutations and multiplications of SNCA lead to an increase in α-synuclein expression and aggregation, which, in turn, leads to increased neuroimmune activation in the brain. Monocytes, macrophages, and microglia (a type of brain cell) play roles in PD, and variants of the gene LRRK2, one of the most common genetic causes of PD, affect cytokine production and inflammatory signaling in these cells. Mutations in GBA1 disrupt lysosomal degradation pathways, leading to increased α-synuclein levels and inflammation [152,153].

In parallel, mutations in PINK1 and PRKN impair mitochondrial quality control and mitophagy, resulting in oxidative stress and immune response activation. In contrast, mutations in DJ-1 decrease the ability to protect cells from oxidative stress and promote inflammatory damage. These results suggest that genetically predisposed individuals may be most affected by the pathogenic mechanism of neuroimmune dysregulation. Table 2 lists some major immune-associated mediators, signaling pathways, and PD-linked genes that play roles in neuroimmune dysfunction, as shown by McWilliams and Muqit (2017) and Truban et al. (2016) [154,155].

### 3.9 Integrated stage-dependent model of neuroinflammation and neuron–immune crosstalk in PD

Current evidence indicates that chronic neuroinflammation arises through a multifactorial process in which α-synuclein aggregation, mitochondrial dysfunction, oxidative stress, BBB dysfunction, peripheral immune activation, and genetic risk all play interconnected roles. Misfolded α-synuclein triggers immune responses by stimulating microglial and astroglial activation, releasing pro-inflammatory cytokines, disrupting cellular clearance pathways, and exacerbating neuronal damage. At the same time, BBB disruption facilitates the infiltration of peripheral immune cells, while mitochondrial dysfunction and inflammasome activation further enhance inflammatory activity [156,157].

Reciprocal interactions between neurons and immune cells establish multiple feed-forward loops that perpetuate neuroimmune dysregulation and progressively compromise dopaminergic neuron survival. This integrated framework underscores the importance of neuron–immune crosstalk as a central contributor to PD progression, rather than as an isolated or universally initiating mechanism. Collectively, these findings provide a strong rationale for therapeutic strategies aimed at restoring immune homeostasis and interrupting pathological inflammatory cascades (Fig. 3, Fig. 4) [12,158].

A fundamental question in PD neuroimmunology is whether neuroinflammation functions primarily as an early contributor to symptom initiation or as an amplifier of disease progression. Current experimental and clinical evidence supports a nuanced, stage-dependent role for neuroinflammation rather than a universal initiating role, although its precise temporal and causal contributions remain unclear, particularly in sporadic PD. Prodromal neuroimaging findings from TSPO-PET studies in LRRK2 mutation carriers and REM-sleep behavior disorder cohorts, together with post-mortem neuropathological analyses at Braak stages I–III, suggest that microglial activation may precede significant dopaminergic neuron loss in selected genetic and prodromal settings, supporting a potential early contributing role in pathological and symptom initiation in susceptible patients. However, these observations do not establish neuroinflammation as a universal or absolute initiating factor in sporadic PD. Conversely, the strongest body of evidence supports neuroinflammation as a self-sustaining disease-modifying process that amplifies neurodegeneration through reciprocal interactions with α-synuclein pathology, mitochondrial dysfunction, oxidative stress, BBB dysfunction, and peripheral immune activation, thereby reinforcing these pathogenic feedback mechanisms. These roles are not mutually exclusive and may overlap and vary with disease stage, genetic background, environmental exposure, and the extent of established neuropathology. Neuroinflammation may contribute to early pathological changes and symptom initiation in genetically susceptible or environmentally exposed individuals, while functioning predominantly as a major amplifier of neuronal injury and disease progression once the pathological neuroimmune feedback circuits are established. This stage-dependent framework has important therapeutic implications, suggesting that early immunomodulatory interventions may attenuate pathological neuroimmune activation in susceptible individuals, whereas interventions during established disease may need to focus on interrupting self-perpetuating inflammatory networks that sustain progressive neurodegeneration. Accordingly, neuroinflammation should be regarded as a context- and stage-dependent contributor to PD pathogenesis rather than as a singular or universally initiating cause.

## 4 Therapeutic targeting of neuron-immune crosstalk

Neuroimmune dysregulation is a key component in the pathogenesis of PD, which has spurred therapeutic efforts beyond mere symptomatic dopamine replacement therapy. Because of the intricate connections between neurons, microglia, astrocytes, peripheral immune cells, and inflammatory mediators, strategies aimed at restoring neuroimmune homeostasis have become exciting strategies for changing the course of PD. All these strategies are designed to reduce chronic inflammation, boost protective immunity, maintain neuronal integrity, and break the self-perpetuating cycles of neurodegeneration. While some methods are still in the preclinical or early clinical stages, they mark a paradigm shift towards treating the mechanisms underlying disease progression, rather than just treating symptoms [159,160].

### 4.1 Modulation of microglial phenotypes

Microglia are key players in regulating neuroinflammatory responses in PD and are among the most studied therapeutic targets. Under physiological conditions, microglia perform immune surveillance, synaptic remodeling, and removal of cellular debris. However, chronic exposure to aggregated α-synuclein and inflammatory stimuli leads to chronic microglial activation and excessive production of cytokines, ROS, and neurotoxic mediators, as shown by Badanjak et al. (2021) [94]. Importantly, microglia in the brains of patients with PD do not constitute a uniform population but comprise heterogeneous transcriptional subgroups with distinct inflammatory, metabolic, and neuroprotective phenotypes.

**Fig. 5:**
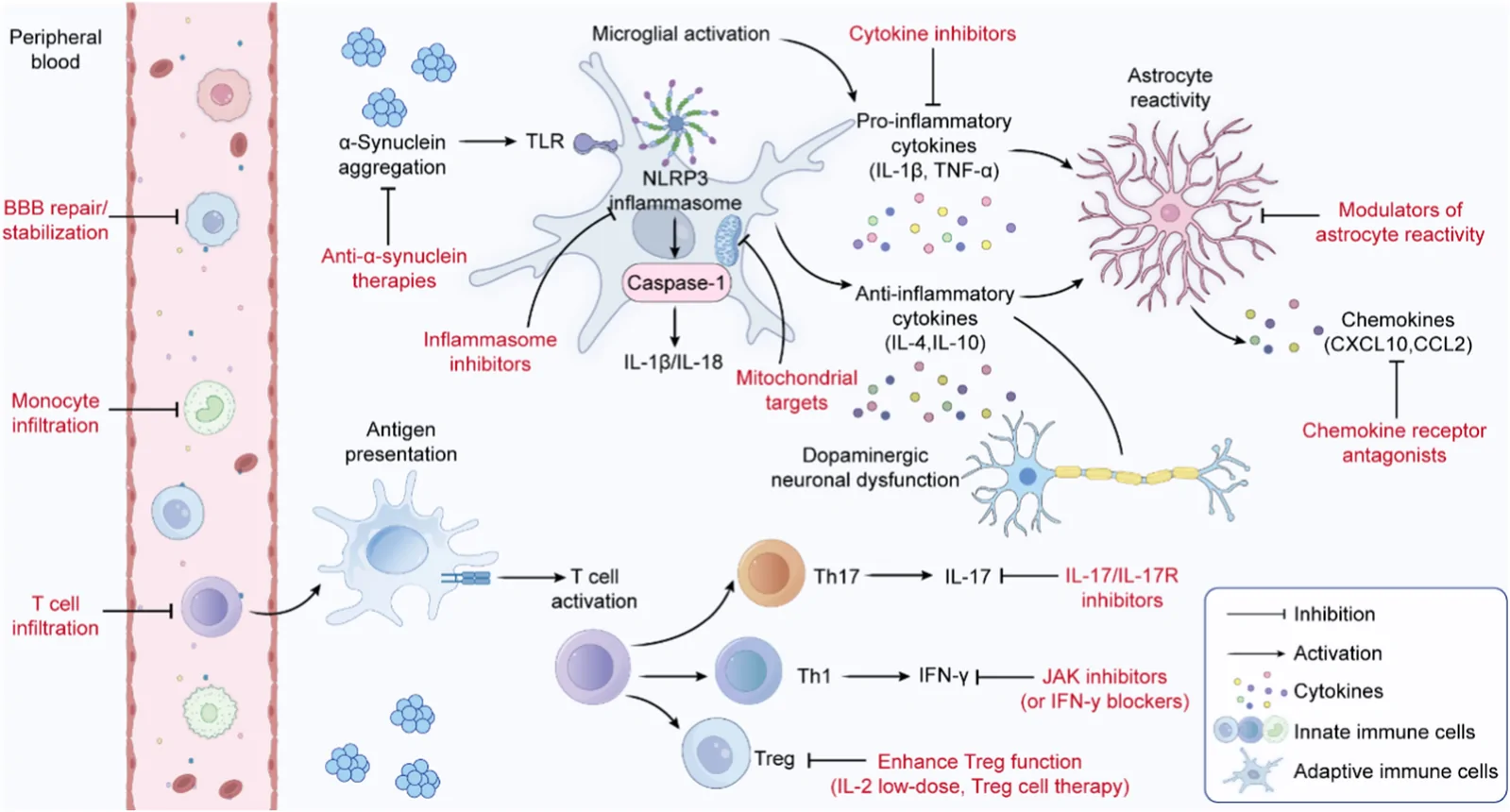
Therapeutic targeting of neuron-immune crosstalk in PD. The major intervention points include α-synuclein aggregation, microglial activation, inflammasome signaling, cytokine and chemokine pathways, astrocyte reactivity, adaptive immune responses, mitochondrial dysfunction, and BBB impairment. Emerging therapeutic strategies aim to restore neuroimmune homeostasis, protect dopaminergic neurons, and slow the disease progression [161]. Created by the authors using Adobe Illustrator.

Current therapeutic approaches focus on regulating the polarization of disease-related microglia towards a neuroprotective phenotype in different transcriptional subgroups, rather than indiscriminately suppressing microglial activity. This subgroup-informed regulatory approach is intended to maintain beneficial immune function without inducing chronic inflammatory response. Evidence suggests that small molecules targeting NF-κB, MAPK, and NLRP3 inflammasome pathways are neuroprotective in experimental models. One of the first microglial modulators studied was minocycline, which inhibits the production of inflammatory cytokines and reduces dopaminergic neuronal loss, while colony-stimulating factor 1 receptor (CSF1R) inhibitors control microglial proliferation and activation. Recently, interventions aimed at microglial metabolism, mitochondrial function, and epigenetic regulation have emerged as novel strategies for restoring immune homeostasis (Fig. 5) [162,163].

### 4.2 Targeting astrocyte-mediated neurotoxicity

Astrocytes play a vital role in maintaining the survival, recycling, and maintenance of synapses and in supporting the BBB. However, chronic exposure to inflammatory stimuli can induce a reactive astrocyte phenotype that disrupts the neuroprotective role and promotes neurodegeneration. Thus, therapies focused on astrocytes have become a complementary approach to neuroimmune modulation in patients with PD [164].

The therapeutic goal is to prevent the formation of neurotoxic astrocytes by blocking inflammatory signals from microglia. Activated microglia release cytokines (such as TNF-α and IL-1α) and complement proteins that are important factors in astrocytic reprogramming. Inhibition of these pathways inhibits astrocyte-mediated neurotoxicity, consistent with preclinical research. A potential strategy for treating neurodegenerative diseases is to restore the homeostatic functions of astrocytes. Increasing glutamate transporter expression can lead to a decrease in excitotoxic neuronal damage, and the activation of neurotrophic factor production by astrocytes, including brain-derived neurotrophic factor (BDNF) and glial cell line-derived neurotrophic factor (GDNF), can promote neuronal survival and repair. As the heterogeneity of astrocytes becomes more apparent, it will be possible to select the target populations of pathogenic astrocytes [165,166].

### 4.3 Cytokine and chemokine-based interventions

Neuroimmune communication involves inflammatory cytokines and chemokines, which are often found in increased amounts in patients with PD. They are important for maintaining neuroinflammation, and attempts have been made to identify therapies targeting neuroinflammation [167].

One of the most well-researched cytokine targets is tumor necrosis factor alpha (TNF-α). Experimental inhibition of TNF-α signaling decreases neuroinflammation, oxidative stress, and the loss of dopaminergic neurons. Blocking the action of IL-1β also reduces inflammasome-mediated inflammatory responses and provides neuroprotection in preclinical models of PD. Other studied cytokines include granulocyte–macrophage colony-stimulating factor, interferon-γ, and IL-6 [168,169].

Chemokine-directed therapies aim to prevent the recruitment of peripheral immune cells to the affected brain regions. Blocking the CCL2, CCR2, CXCL10, and CXCR3 pathways can reduce the infiltration of monocytes and T lymphocytes, as well as decrease inflammatory amplification. In contrast, pro-inflammatory cytokines can be suppressed to encourage immune resolution and repair of tissues. Therapeutic interventions targeting neuroimmune mechanisms in PD are summarized in Table 3 [183,184].

**Table 3:** Current and emerging neuroimmune-targeted therapies for PD.

Therapeutic Category	Representative Targets	Mechanism of Action	Development Stage	References
Microglial modulators	Minocycline, CSF1R inhibitors	Regulating the polarization of disease-related microglia towards a neuroprotective phenotype in different transcriptional subgroups.	Preclinical-Clinical	[170,171]
Inflammasome inhibitors	NLRP3 inhibitors, caspase-1 inhibitors	Reduce IL-1β/IL-18 maturation and neuroinflammation	Preclinical	[172]
Cytokine-targeted therapies	TNF-α, IL-1β, and IL-6 antagonists	Block pro-inflammatory signaling cascades	Experimental	[173]
Chemokine inhibitors	CCR2, CXCR3 antagonists	Limit recruitment of peripheral immune cells	Preclinical	[174]
Treg-based therapies	Low-dose IL-2, adoptive Treg transfer	Restore immune tolerance and suppress inflammation	Preclinical	[175]
Immune checkpoint modulators	PD-1/PD-L1, CTLA-4 pathways	Rebalance adaptive immune responses	Emerging	[176]
α-Synuclein immunotherapies	Vaccines, monoclonal antibodies	Promote aggregate clearance and reduce antigen burden	Clinical evaluation	[177]
Astrocyte-directed therapies	Anti-TNF-α, anti-IL-1α approaches	Prevent neurotoxic astrocyte transformation	Preclinical	[99]
Mitochondrial-targeted therapies	CoQ10 derivatives, mitophagy enhancers	Reduce oxidative stress and improve mitochondrial function	Preclinical–Clinical	[178]
BBB-protective agents	VEGF/MMP modulators, endothelial stabilizers	Restore neurovascular integrity and reduce immune infiltration	Preclinical	[179]
Gene-based therapies	SNCA, LRRK2, GBA1 targets	Modify disease-driving molecular pathways	Emerging	[180]
Combination neuroimmune therapies	Multi-target immunomodulatory approaches	Simultaneous targeting of protein aggregation, inflammation, and neurodegeneration is essential.	Emerging	[181]
Therapeutic Category	Representative Targets	Mechanism of Action	Development Stage	[182]

Although experimental therapies with cytokines are promising, there are many challenges in translating these therapies into clinical use, such as systemic immunosuppression, poor BBB penetration, and complex and interconnected inflammatory networks [185].

### 4.4 Adaptive immune modulation and immune checkpoints

With the increasing understanding of the involvement of adaptive immunity in PD, new opportunities for potential therapeutic interventions have emerged. There are increasing reports indicating that antigen-specific T-cell responses against α-synuclein play a role in the progression of the disease through chronic neuroinflammation and neuronal damage [186].

A potentially useful approach is to improve the activity of Tregs, which play a key role in maintaining immune tolerance and preventing exaggerated inflammatory responses from the immune system. Experimental models have shown that the number of Tregs in the brain decreases with an increase in neuroinflammation and degeneration of dopaminergic neurons. Transplantation of Tregs or adoptive transfer of these cells can reduce neuroinflammation and protect dopaminergic neurons. Thus, there is a great deal of current research on pharmacological agents that promote Treg differentiation and function [72,187].

Another promising pathway is the immune checkpoint pathway. Programmed cell death protein 1 (PD-1), programmed death ligand-1 (PD-L1), and cytotoxic T-lymphocyte-associated protein-4 (CTLA-4) are molecules that control immune activation and tolerance. Modulation of these pathways may play a role in restoring immune balance and reducing chronic inflammation. Moreover, α-synuclein-specific vaccines and passive immunotherapy using monoclonal antibodies target α-synuclein aggregates to promote clearance while minimizing antigen-induced activation of the immune system (Fig. 5). A few candidates, including those identified by Dey et al. (2024) and Meissner et al. (2020), have undergone clinical evaluation, demonstrating the translational potential of adaptive immune modulation [188,189].

### 4.5 Restoration of BBB integrity

There is growing awareness that BBB dysfunction may be a result of and a contributing factor to PD neuroinflammation. A break in barrier integrity allows peripheral immune cells and circulating inflammatory mediators to exacerbate neuronal injury via neuroimmune activation [22].

Therapeutic approaches for BBB dysfunction include repair, reinforcement, and anti-inflammatory approaches. The modulation of vascular endothelial growth factor (VEGF), matrix metalloproteinases (MMPs), and inflammatory cytokines has shown promise in enhancing barrier stability. Maintenance of pericyte function and astrocytic support in the neurovascular unit could also further strengthen BBB integrity and help minimize immune cell infiltration [71,190].

Significantly, restoration of BBB integrity may offer advantages other than inflammation control. An enhanced barrier function may minimize exposure to systemically acting toxins, normalize CNS homeostasis, and increase the effectiveness of other disease-modifying treatments. Hence, neurovascular protection is considered crucial in the overall treatment of PD [22,142].

### 4.6 Emerging disease-modifying neuroimmune therapeutics

Given the multifactorial nature of PD, interventions aimed at disease-modifying effects are likely to involve multiple pathological processes. New therapeutic strategies are based on increasingly integrated strategies targeting α-synuclein aggregation and associated neuroinflammation, oxidative stress, mitochondrial dysfunction, and immune dysregulation [182]. Throughout this review, the term 'disease modification' refers strictly to interventions that demonstrably alter the underlying pathophysiological trajectory of PD, defined as slowing, halting, or reversing dopaminergic neuron loss and associated functional decline, as distinct from purely symptomatic relief. Preclinical evidence of reduced α-synuclein aggregation, microglial activation, or oxidative stress represents surrogate endpoint data and does not, by itself, constitute functional disease modification. We explicitly note where evidence is limited to surrogate pathological markers and where functional or behavioral endpoints have been demonstrated. This distinction is particularly important for interpreting nanoplatform studies discussed in Section 7, where most current evidence is preclinical and functional validation remains an important unmet benchmark.

Given the pivotal role of NLRP3 signaling in neuroimmune activation in the brain, inflammatory inhibitors have been the focus of several studies. Likewise, other drugs targeting mitochondria focus on decreasing oxidative stress, enhancing mitophagy, and mitigating inflammatory amplification that can result from cell energy failure. Direct modulation of disease-driving pathways may be achieved using gene-based therapies directed at genes associated with PD, such as SNCA, LRRK2, GBA1, and more [191].

Emerging multimodal therapeutic strategies combine multiple mechanisms, including neuroimmune modulation, α-synuclein clearance, neurotrophic support, regenerative therapies, and BBB stabilization. With the development of new biomarkers and precision medicine, it may be possible to stratify patients based on their individual inflammatory and molecular profiles, allowing tailored therapies for patients with PD. Existing and new therapeutic strategies focusing on the neuroimmune axis and level of development are summarized in Table 3. These advances highlight the increasing promise of neuroimmune-directed therapies in changing the nature of PD from symptom management to disease modification (Fig. 5) [192,193].

## 5 Nanoplatform technologies for PD intervention

**Fig. 6:**
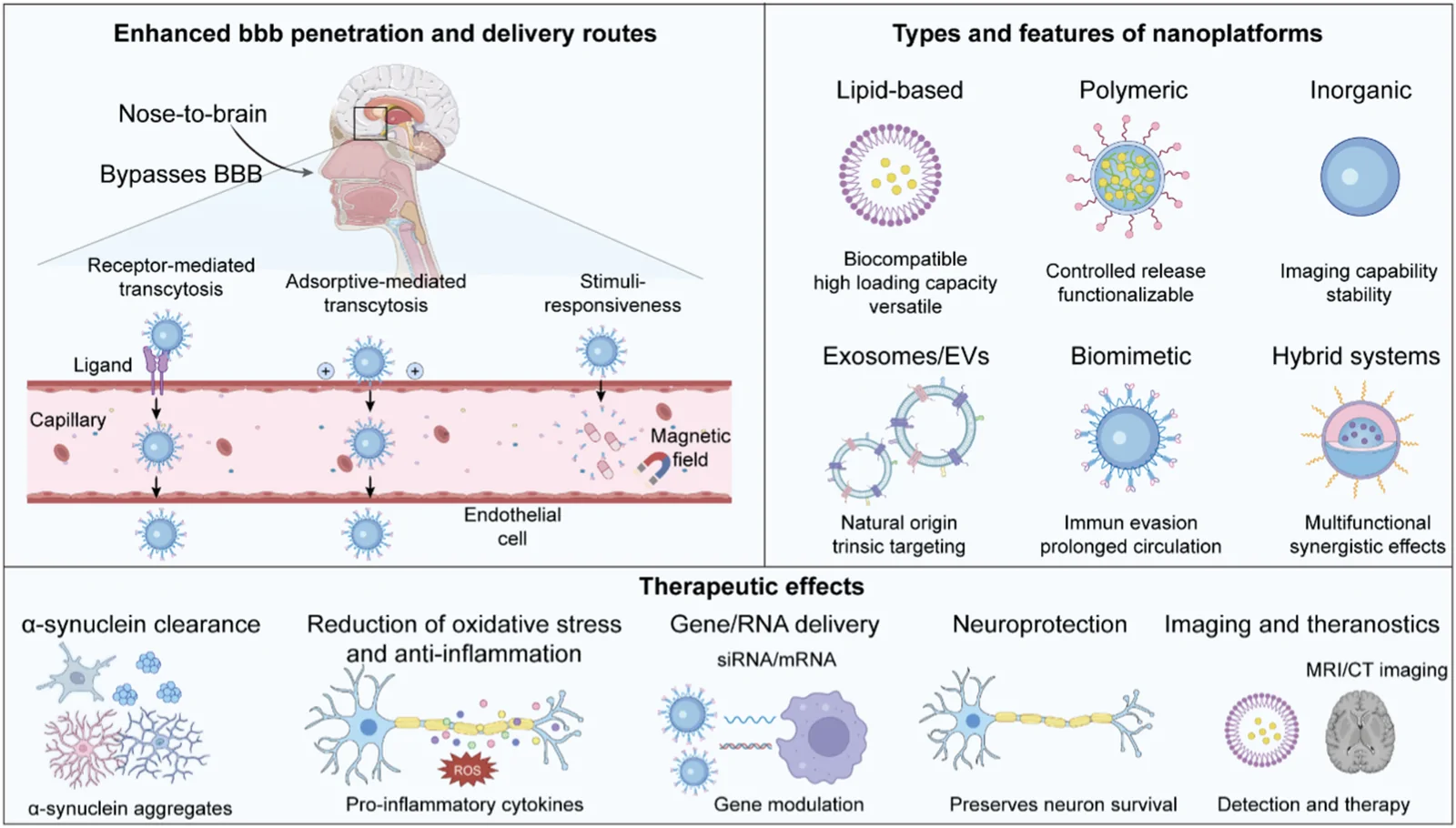
Schematic overview of lipid-based nanocarriers, polymeric nanoparticles, inorganic nanomaterials, hybrid nanoplatforms, biomimetic nanoparticles, extracellular vesicles (EVs), and stimuli-responsive nanosystems, illustrating their transport across biological barriers and their applications in neuroprotection, α-synuclein clearance, gene and RNA delivery, immunomodulation, and theranostic imaging in Parkinson's [195,196].

Because PD has a multifactorial character (α-synuclein aggregation, neuroinflammation, mitochondrial dysfunction, oxidative stress, BBB impairment, and progressive neuronal loss), there are major difficulties in treating it with conventional therapies. Many compounds have been proven to be effective in experimental models. However, their clinical efficacy is hindered by several factors, including poor pharmacokinetics, inability to penetrate the BBB, systemic toxicity, and low target specificity [194]. Nanotechnology has become a game-changing platform that may break these barriers by developing engineered structures on the nanoscale that can be designed to deliver therapeutics into target regions with better bioavailability, penetrate the BBB, and target pathological processes in the brain microenvironment. Nanomedicine has enabled the development of therapeutic delivery and modulation of several disease pathways (Fig. 6) for the delivery of drugs, proteins, nucleic acids, and regenerative factors. Therefore, nanotechnology is increasingly viewed as a key element of next-generation disease-modifying drugs for PD, as demonstrated by Cheng et al. (2022) [197].

### 5.1 Rationale for nanomedicine in neurodegenerative disorders

The highly protected environment of the CNS makes neurodegenerative diseases particularly difficult to treat. Pharmacological treatments are often ineffective in reaching therapeutic levels in tissues because of their low solubility, rapid metabolism, low cellular uptake, and limited BBB permeability [198].

Nanomedicine has several benefits that directly overcome these limitations. The nanosize of nanocarriers allows them to interact efficiently with biological membranes, be delivered into cells, and enhance biodistribution. Packaging in nanoparticles prevents the enzymatic degradation of therapeutic agents and enhances their bioavailability and circulation times. Moreover, nanocarriers can be designed to allow controlled and sustained drug release, thus limiting systemic exposure and side effects [199].

One of the main benefits of nanotechnology is its multifunctionality. In contrast to conventional drugs, which are usually designed to target a single pathway, nanoplatforms can carry multiple drugs and/or be used for diagnosis and therapy on the same platform. In PD, disease processes are complex and are driven by a combination of pathological processes that must be addressed in a coordinated manner, making such capabilities extremely useful [200].

### 5.2 Biological barriers limiting therapeutic delivery

For effective therapy of PD, the efficient delivery of therapeutics to the affected brain regions is key. However, several barriers are present in the brain to prevent drugs from reaching the brain tissue and reduce their effectiveness [201].

The biggest challenge remains BBB permeability. The BBB is composed of endothelial cells with tight junctions, astrocytes, and pericytes that tightly control the movement of molecules between the blood and CNS. Owing to its highly selective permeability, approximately 98% of small-molecule drugs and nearly all biologics exhibit poor BBB penetration, preventing many potentially beneficial neuroprotective agents from reaching therapeutically effective concentrations within the brain [202]. Critically, a quantitative summary analysis encompassing more than 200 published studies of intravenously administered nanocarriers reported a median brain delivery efficiency of only 0.65% of the injected dose (%ID) [203,204]. Across the analyzed studies, even actively targeted nanoparticles achieved brain parenchymal uptake of less than 0.1-1% ID. Because this value was derived from a pooled analysis of more than 200 published nanocarrier studies rather than from a single formulation or experimental model, it should be interpreted as an aggregate benchmark for the field and not as a universally applicable delivery efficiency for individual nanocarrier systems. This fundamental quantitative limitation underscores the pressing need for alternative routes of administration (e.g., intranasal delivery, focused ultrasound-assisted BBB opening), transcytosis-enhancing surface modifications, and cell-mediated delivery strategies to achieve therapeutically relevant brain concentrations.

Other factors that limit the use of these substances include enzymatic breakdown in the bloodstream, rapid renal clearance, uptake by the reticuloendothelial system, poor ability to cross the brain extracellular space, and poor cellular uptake of these substances. Proteins, peptides, antibodies, and nucleic acids are large biomolecules that are subject to harsh delivery conditions owing to their size and instability [205].

**Fig. 7:**
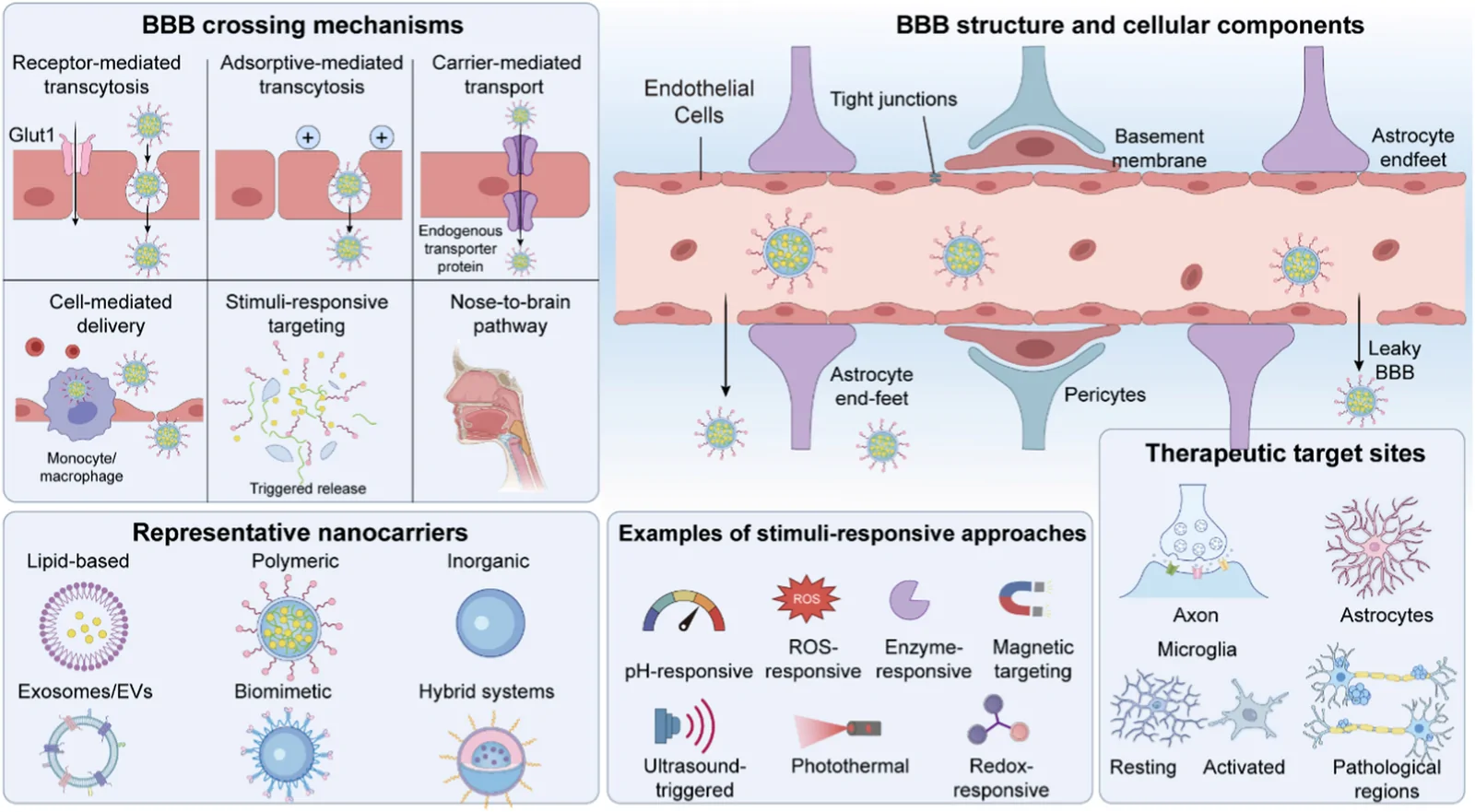
BBB penetration and brain-targeting mechanisms of nanocarriers. Schematic illustration of receptor-mediated transcytosis, adsorptive-mediated transcytosis, carrier-mediated transport, cell-mediated delivery, intranasal nose-to-brain transport, and stimuli-responsive targeting strategies that enhance therapeutic delivery to the CNS [206].

This heterogeneous disease pathology complicates therapeutic delivery, as effective therapies may need to selectively target neurons, microglia, astrocytes, and other relevant cell populations within specific brain regions. Nanotechnology offers a universal solution to these issues through several BBB-crossing and brain-targeting mechanisms (Fig. 7) [207]. The marker should be replaced with the citation number of the original quantitative analysis supporting both the 0.65% injected-dose value and the sample of more than 200 published studies [208,209].

### 5.3 Design principles of brain-targeted nanoplatforms

The physicochemical characteristics of nanocarriers influence their therapeutic effectiveness. To design rational nanoplatforms, it is important to optimize the particle size, morphology, and surface charge, in addition to optimizing the particle composition, biodegradability, capacity for loading therapeutic agents, and targeting functionality [210].

Biodistribution and BBB penetration are greatly affected by particle size. The circulation profile and tissue penetration of nanoparticles with sizes between approximately 10 and 200 nm are generally good. Surface charge also affects biological interactions; a surface with a moderately neutral or slightly negative surface charge may exhibit better biocompatibility and less immune recognition [211].

Surface functionalization is one of the most crucial design approaches for brain-targeted delivery. Ligands that bind to receptors on BBB endothelial cells, such as transferrin, insulin, and low-density lipoprotein (LDL) receptors, can be attached to nanoparticles. These changes aid receptor-mediated transcytosis and brain accumulation. Moreover, transport engineering allows nanocarriers to exploit receptor-, adsorptive-, carrier-, and cell-mediated delivery routes to improve CNS targeting (Fig. 7) [212].

Long-term toxicity and tissue deposition are mostly avoided by using biodegradable materials in clinical applications. In addition, the presence of responsive elements allows for the controlled release of drugs when stimulated by pathological factors such as oxidative stress, acidic pH, inflammatory enzymes, and external factors [213].

### 5.4 Lipid-based nanocarriers

Patravale et al. (2018) reported that lipid-based nanocarriers are among the most advanced and clinically ready nanotechnology platforms available. They have good biocompatibility because of their high structural similarity to biological membranes and are easily taken up by cells [214].

Liposomes are lipid vesicles that can encapsulate both hydrophilic and hydrophobic therapeutics. They have been used to deliver a wide variety of molecules, including dopamine agonists, antioxidants, anti-inflammatory agents, and nucleic acids to experimental models of PD. Enhanced BBB penetration and neuronal uptake can also be achieved by surface modification with targeting ligands [215].

Conventional liposomes, which are difficult to maintain, are more stable when solid lipid nanoparticles (SLNs) are used, as they have favorable biocompatibility profiles. Nanostructured lipid carriers (NLCs) are second-generation lipid systems that consist of a combination of solid and liquid lipids to enhance the drug loading capacity and minimize early drug release [216].

In recent years, LNPs have garnered intense interest as effective carriers for nucleic acid delivery. Such systems hold great promise for delivering small interfering RNAs (siRNAs), messenger RNA (mRNA), and components for gene editing into specific genes associated with PD, such as SNCA and LRRK2. The main classes of nanoplatforms, their properties, advantages, disadvantages, and some representative applications in PD are briefly summarized in tabular form in Table 4 [230]. Compared with polymeric nanoparticles, LNPs offer superior nucleic acid encapsulation efficiency and benefit from the most advanced clinical validation (having underpinned COVID-19 mRNA vaccines), but present greater cold-chain storage requirements and manufacturing complexity that may limit their accessibility for CNS applications.

**Table 4:** Comparison of major nanoplatform classes for PD therapy, including their composition, advantages, limitations, and representative therapeutic applications.

Nanoplatform Class	Major Components	Key Advantages	Major Limitations	Representative Applications in PD	References
Liposomes	Phospholipid bilayers	Excellent biocompatibility; capable of loading both hydrophilic and hydrophobic drugs	Limited physical stability; potential drug leakage	Dopamine agonists, antioxidants, anti-inflammatory agents, nucleic acid delivery	[215]
SLNs	Solid lipid matrices	Improved stability; controlled drug release; good biocompatibility	Lower drug-loading capacity compared with newer lipid systems	Neuroprotective drug delivery and sustained release formulations	[217]
NLCs	Mixture of solid and liquid lipids	Higher loading efficiency; reduced drug leakage; enhanced stability	More complex formulation and manufacturing	Dopamine replacement therapies and antioxidant delivery	[218]
LNPs	Ionizable lipids, phospholipids, cholesterol, PEG-lipids	Highly efficient nucleic acid delivery; clinically validated platform	Manufacturing complexity and storage requirements	siRNA, mRNA, and gene-editing therapeutics targeting SNCA and LRRK2	[219]
PLGA Nanoparticles	PLGA polymers	Biodegradable; sustained drug release; regulatory approval history	Initial burst release and limited loading of some biologics	Dopamine, antioxidants, neurotrophic factors, and anti-inflammatory drugs	[220]
Chitosan Nanoparticles	Natural polysaccharide-based polymers	Mucoadhesive; enhanced permeability; suitable for intranasal delivery	Variable stability and batch-to-batch variability	Nose-to-brain drug delivery and neuroprotective therapies	[221]
Dendrimers and Polymeric Micelles	Branched polymers and amphiphilic copolymers	High functionalization capacity; controlled release; gene delivery potential	Potential toxicity at high concentrations	Gene delivery, immunomodulation, and targeted drug transport	[222]
Gold Nanoparticles	Metallic gold nanostructures	Easy surface functionalization; imaging and theranostic capabilities	Potential long-term accumulation and clearance concerns	Theranostics, targeted drug delivery, and imaging applications	[223]
Iron Oxide Nanoparticles	Magnetic iron oxide cores	MRI visibility; magnetic targeting; image-guided delivery	Aggregation and potential magnetic-related toxicity	Magnetic targeting and diagnostic imaging	[224]
Cerium Oxide Nanoparticles	Redox-active inorganic nanomaterials	Intrinsic antioxidant activity; ROS scavenging capability	Limited clinical validation and long-term safety data	Reduction of oxidative stress and neuroprotection	[225]
Biomimetic Nanoparticles	Cell membrane-coated nanoparticles (RBC, macrophage, stem-cell membranes)	Immune evasion; prolonged circulation; enhanced targeting	Complex fabrication and scalability challenges	Neuroinflammation targeting and BBB navigation	[226]
Exosomes/EVs	Naturally secreted biological vesicles	Excellent BBB penetration; low immunogenicity; natural targeting	Difficult large-scale production and standardization	RNA delivery, neuroimmune modulation, and neurotrophic factor transport	[227]
Hybrid Nanoparticles	Lipid–polymer or lipid–inorganic combinations	Multifunctionality; synergistic therapeutic and imaging capabilities	Manufacturing and characterization complexity	Combined drug delivery, imaging, and neuroprotection	[228]
Stimuli-Responsive Nanoplatforms	Smart materials responsive to pH, ROS, enzymes, magnetic fields, ultrasound, or light	On-demand and site-specific drug release; improved precision	Design complexity and regulatory challenges	Precision neurotherapeutics and controlled therapeutic delivery	[229]

### 5.5 Polymeric nanoparticles

Kadam et al. (2024) showed that polymeric nanoparticles are one of the most widely studied nanocarriers used for drug delivery to the CNS due to their versatility, biodegradability, and tunable physicochemical properties. Natural or synthetic polymers can be used to create these systems with well-defined drug-release profiles [231].

The most commonly used material is poly(lactic-co-glycolic acid) (PLGA), which has a good safety record and is well accepted for use. PLGA nanoparticles have been used to deliver various neurotransmitters, neurotrophic factors, antioxidants, anti-inflammatory agents, and gene therapeutics to the brain. Their degradation is controlled, allowing prolonged drug release and therapeutic effects [232].

Compared to other nanoparticles, chitosan-based nanoparticles have other benefits that make them more interesting for intranasal delivery strategies, such as their mucoadhesive properties and enhanced permeability across biological membranes. Several other polymeric systems, such as polyethyleneimine, polycaprolactone, dendrimers, and polymeric micelles, have been found to be useful for gene delivery, neuroprotection, and immunomodulatory applications [233]. Relative to lipid-based nanocarriers, PLGA and chitosan nanoparticles offer superior physicochemical tunability and room-temperature stability, but typically exhibit lower nucleic acid encapsulation efficiency and require more complex surface engineering to achieve comparable BBB penetration rates.

### 5.6 Inorganic and hybrid nanomaterials

Inorganic nanomaterials have unique physicochemical characteristics and properties that can be used for therapeutic and diagnostic purposes. Gold nanoparticles, iron oxide nanoparticles, silica nanoparticles, cerium oxide nanoparticles, and quantum dots have been investigated for neurological applications [234].

Gold nanoparticles (AuNPs) are highly biocompatible, surface-modifiable imaging agents that are beneficial for theranostics. Iron oxide nanoparticles can be used for magnetic targeting, magnetic resonance imaging, and drug delivery.

In the inorganic category, cerium oxide nanoparticles are noteworthy due to their intrinsic antioxidant properties. They can repeatedly remove ROS through reversible redox cycling to reduce oxidative stress, a key factor in the pathogenesis of dopaminergic neurodegeneration. Hybrid nanomaterials are composed of organic and inorganic materials that can integrate complementary functions, such as controlled drug release, imaging, antioxidant activity, and targeted delivery, on a single platform [235,236]. While inorganic nanomaterials such as cerium oxide and iron oxide nanoparticles offer unique intrinsic catalytic or magnetic functionalities unavailable to organic carriers, their slower biodegradation kinetics and potential long-term accumulation in the liver and spleen present greater safety evaluation requirements compared with biodegradable lipid or polymer platforms.

### 5.7 Biomimetic nanoplatforms

Biomimetic nanotechnology imitates natural forms to increase compatibility with the physiological system. These platforms exploit endogenous transport mechanisms and simultaneously minimize drug clearance by the immune system before reaching their target [237].

Nanoparticles coated with cell membranes are very interesting and promising biomimetic systems. Macrophage-, leukocyte-, erythrocyte-, platelet-, and stem cell-derived membranes can 'cloak' synthetic nanoparticles, allowing them to circulate longer in the body and target inflamed tissues more effectively. Inflammation of the nervous system is a hallmark of PD, and immune cell-derived membranes can promote preferential deposition in the affected brain regions [201].

Biomimetic nanocarriers also exhibit improved interactions with biological barriers and low immune activation in the host. Exosomes are promising targets for therapies designed to target the neuroimmune system because they can mimic the natural pathways of cellular communication [238].

### 5.8 Exosome and extracellular vesicle-based systems

Exosomes and EVs are novel nanoplatforms with great potential for neurological applications. Vesicles are naturally occurring and secreted by almost all cell types and act as endogenous mediators of intercellular communication. Exosomes are an ideal therapeutic option for PD for several reasons: they are highly biocompatible, have low immunogenicity, possess intrinsic BBB-penetrating properties, and have natural targeting properties. Exosomes can carry proteins, lipids, mRNA, microRNA, siRNA, and gene-editing cargoes, shielding them from degradation. In experimental PD models, anti-inflammatory molecules, neurotrophic factors, antioxidant enzymes, and RNA therapeutics have been delivered using engineered exosomes [201].

In addition, exosomes are integral to neuron-immune communication and provide a natural opportunity to target pathological interactions between neurons and immune cells in patients with PD. Brain-targeted delivery systems (Fig. 7) are especially appealing because of the endogenously existing BBB-crossing systems with which they are compatible. Issues related to large-scale production, purification, and standardization remain major challenges but are slowly being overcome with the development of bioengineering and manufacturing technologies [239]. Exosomes and EVs offer the unique advantages of inherent BBB-crossing capability and ultra-low immunogenicity compared with synthetic nanocarriers; however, their clinical translation is currently impeded by the lack of scalable GMP-compliant manufacturing protocols and reliable quality control standards to ensure batch consistency.

### 5.9 Stimuli-responsive and multifunctional nanoplatforms

Chen et al. (2024) reported that the next generation of nanomedicine involves intelligent multifunctional systems that can react dynamically to pathological microenvironments. Stimuli-responsive nanoplatforms are designed to selectively deliver therapeutic cargo to target tissues under specific conditions [240].

Endogenous triggers include oxidative stress, acidity, inflammatory enzymes, elevated ROS levels, and metabolites associated with diseases. Anti-inflammatory or antioxidant agents, for instance, can be released from nanoparticles in response to ROS only in inflamed tissues, where they have the most therapeutic effect with minimal off-target effects [241].

Drug release and targeting can be further controlled using external stimuli, such as magnetic fields, ultrasound, temperature variations, and light irradiation. For example, magnetic nanoparticles can be targeted to specific regions of the brain using an external magnetic field.

Multifunctional nanoplatforms combine the following functions in a single platform: imaging, targeted delivery, controlled release, immunomodulation, neuroprotection, gene delivery, and regenerative support. These theranostic platforms are of great interest in PD, as they are involved in the multifactorial disease process and allow real-time monitoring of therapeutic responses. A comparative summary of the investigated nanoplatforms is presented in Table 4. With the ongoing development of nanotechnology, multifunctional and personalized nanotherapeutics will play an increasingly important role in developing disease-modifying interventions for Parkinson's disease (Fig. 6) [242].

## 6 Nanoplatform-mediated neuroprotection through immune modulation

**Fig. 8:**
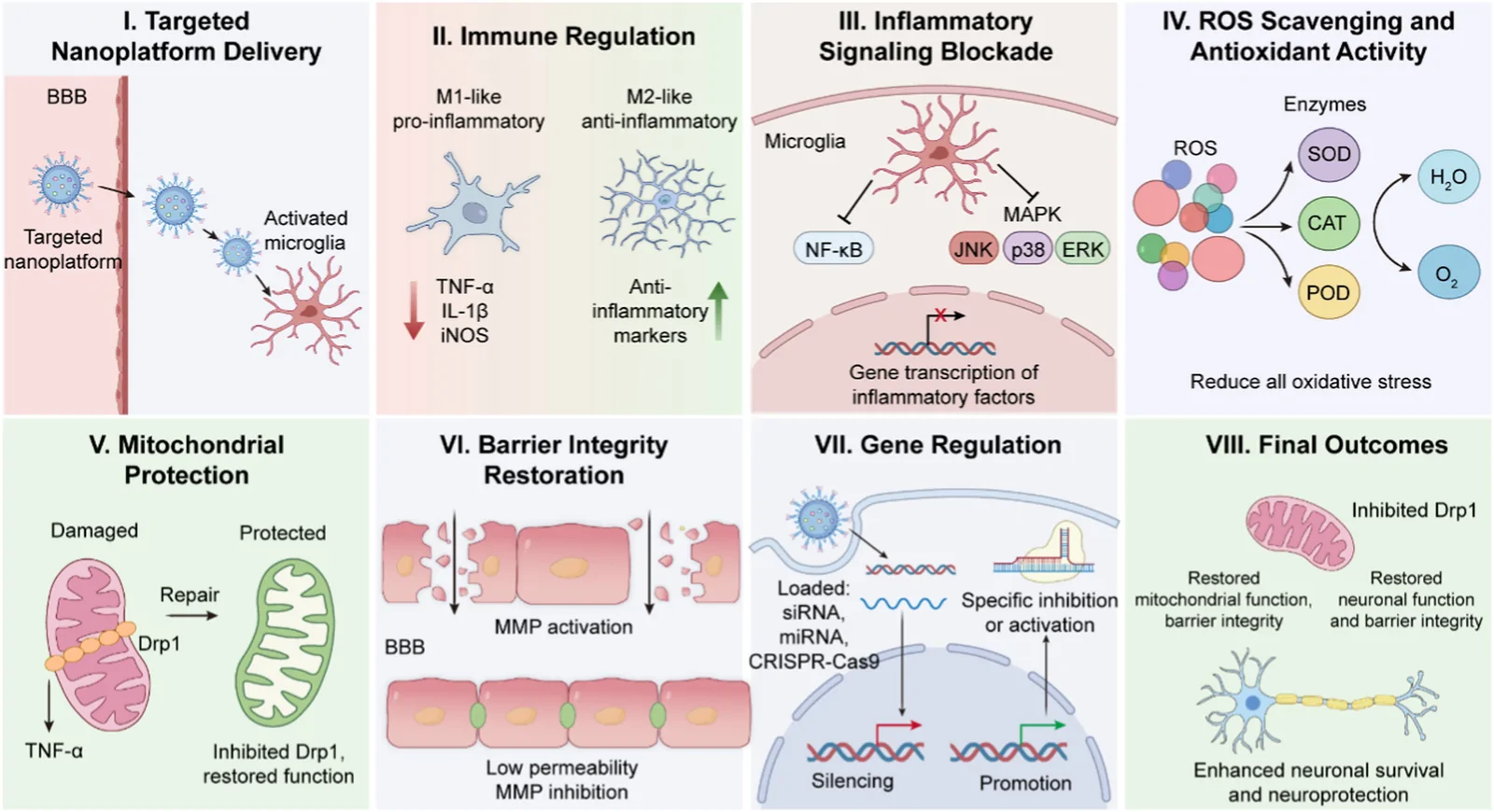
Nanoplatform-mediated neuroprotection via immune regulation in PD. Nanocarriers deliver anti-inflammatory agents, antioxidants, nucleic acids, and gene-regulatory therapeutics across the BBB to modulate microglial activation, suppress inflammatory signaling pathways, reduce oxidative stress, protect mitochondrial function, restore barrier integrity, and enhance neuronal survival [243]. Created by the authors using Adobe Illustrator.

Neuroinflammation plays a significant role in the onset and development of PD, and immune modulation is an attractive therapeutic approach for treating it. However, many anti-inflammatory and neuroprotective drugs are poorly permeable to the BBB, easily degraded, not target-specific, and have side effects in peripheral tissues. Targeted delivery, controlled release, and enhanced bioavailability are some of the key challenges that nanotechnology can address. Many nanoplatforms can not only act as passive drug transporters but also have active immunoregulatory, antioxidant, mitochondrial, and gene expression-modulating activities. Thus, nanomedicine has become a novel strategy to prevent the degeneration of vulnerable dopaminergic neurons and simultaneously modulate the neuroimmune mechanisms that promote disease activity in various ways, as reported by Ahmad et al. (2024) and Kaur et al. (2025) (Fig. 8) [201,244].

### 6.1 Targeting microglial activation pathways

Microglial activation is one of the most common and long-lasting neuroimmune changes observed in PD. Persistent activation by aggregated α-synuclein, damage-associated signals from mitochondria, and inflammatory cytokines results in the release of cytokines, ROS, and nitric oxide, all of which play a role in the progressive degeneration of neurons [90].

Activated microglia have higher phagocytic activity and are selectively targeted by nanoparticles, which can be used for the targeted delivery of anti-inflammatory compounds. Suppression of key inflammatory pathways, such as NF-κB, MAPK, and NLRP3 inflammasome signaling, has been achieved with the delivery of agents using LNPs, polymeric nanocarriers, exosomes, and biomimetic systems. These therapies lower the levels of the cytokines TNF-α, IL-1β, and IL-6 and restore microglial homeostatic functions [245].

In experimental PD models, several studies have shown that curcumin nanoparticles inhibit microglial activation and reduce the secretion of inflammatory cytokines. Similarly, resveratrol-loaded nanocarriers can block the response of microglial cells triggered by oxidative stress and improve neuronal survival. Increased local drug concentrations in the affected brain regions, achieved through nanoparticle-mediated delivery of anti-inflammatory peptides and immunomodulatory molecules, further enhance therapeutic efficacy [246]. Mechanistically, nanoparticle-mediated suppression of NF-κB and MAPK signaling promotes a phenotypic shift of pro-inflammatory M1-like microglia (characterized by iNOS, TNF-α, and IL-6 upregulation) toward neuroprotective M2-like states (characterized by Arg-1, CD206, and IL-10 expression), restoring phagocytic clearance of α-synuclein without the collateral neurotoxic effects of sustained inflammatory activation.

Macrophage/leukocyte membrane-coated emerging biomimetic nanoplatforms exploit endogenous inflammatory homing mechanisms, allowing preferential accumulation at neuroinflammatory sites. These are more specific therapies that can be used to achieve the most therapeutic effect with minimal systemic immunosuppression [247].

### 6.2 Suppression of neuroinflammatory cascades

Microglial activation is not the only process of neuroinflammation in PD; astrocytes, infiltrating immune cells, endothelial cells, and neurons are also involved in neuroinflammation. These cellular interactions result in complex inflammatory cascades involving the amplification of cytokines, chemokine production, and continuous immune activation [248].

Nanoplatforms can target multiple components of inflammatory networks. Controlled-release systems can deliver anti-inflammatory therapeutics over an extended period to maintain the suppression of pathogenic pathways and minimize concentration fluctuations within the bloodstream. Corticosteroids, cytokine inhibitors, natural anti-inflammatory compounds, and other immunomodulatory agents have been delivered using nanocarriers, leading to increased bioavailability in the brain [249].

The focus has been specifically on NF-κB signaling, which regulates the expression of inflammation-related genes. In experimental models of neurodegenerative diseases, NF-κB inhibitors can be delivered via nanoparticles to decrease the secretion of proinflammatory cytokines and suppress neurodegeneration. Similarly, nanocarriers that inhibit the activation of the NLRP3 inflammasome inhibit the maturation of IL-1β and break inflammatory amplification loops. Thus, nanoplatforms may block NF-κB/MAPK signaling, decrease the production of cytokines, and change the activation states of glia, thereby disrupting neuronal inflammatory cascades (Fig. 8). Zhao et al. (2025) showed that multifunctional nanoplatforms, such as anti-inflammatory agents, antioxidants, neurotrophic factors, and gene therapeutics, are particularly important [250]. Beyond innate immune modulation, certain nanoplatforms also interface with adaptive immunity by promoting regulatory T cell (Treg) responses. Nanoparticle-delivered TGF-β and IL-2 payloads, as well as tolerogenic antigen presentation facilitated by biomimetic nanocarriers, may support Treg expansion and function in the PD brain, partially restoring the immune tolerance that is diminished in PD patients.

### 6.3 Antioxidant and mitochondria-protective nanotherapies

Mitochondrial dysfunction is a key player in the vulnerability of dopaminergic neurons in PD, and oxidative stress is a key factor in this dysfunction. Overproduction of ROS causes damage to proteins, lipids, and nucleic acids, as well as α-synuclein aggregation and brain inflammation. Antioxidants can exert significant neuroprotective effects, as oxidative stress and neuroinflammation mutually reinforce each other [251].

Nanotechnology offers myriad opportunities for the use of antioxidant compounds in therapeutic applications by increasing compound stability, bioavailability, and tissue targeting. The use of nanocarriers to entrap antioxidants, such as curcumin, resveratrol, coenzyme Q10, quercetin, and epigallocatechin gallate, increases BBB penetration and extends their therapeutic efficacy [252].

Some nanomaterials with intrinsic antioxidant properties can be used as antioxidant delivery agents. One such example is cerium oxide nanoparticles. These nanoparticles exert catalytic antioxidant activity through reversible Ce^3+^/Ce^4+^ redox cycling: Ce^3+^ reduces superoxide radicals (O_2_•^-^) to hydrogen peroxide (SOD-mimetic activity), while Ce^4+^ subsequently decomposes hydrogen peroxide to water and oxygen (CAT-mimetic activity), regenerating Ce^3+^ in the process. This catalytic cycle is non-consumptive, enabling sustained multi-turnover ROS scavenging without depletion of the nanoparticle itself [253]. Selenium nanoparticles function as glutathione peroxidase (GPx) mimetics via a selenol (-SeH) active center: the selenol group catalyses the reduction of hydroperoxides (ROOH) coupled to GSH oxidation (2 GSH + ROOH → GSSG + H_2_O + ROH), thereby restoring intracellular redox homeostasis and attenuating lipid peroxidation-driven dopaminergic injury.

An increasing number of therapeutic approaches involve the direct targeting of mitochondria. Mitochondria-targeted nanoparticles can specifically target damaged organelles for the delivery of antioxidants, respiratory chain stabilizers, and mitochondrial protective peptides. These strategies are useful for maintaining energy production, minimizing ROS production, and decreasing the activation of inflammatory signaling pathways. In addition to immune regulation, some nanotherapeutic systems specifically address other major factors of dopaminergic neurodegeneration, such as oxidative stress and mitochondrial dysfunction (Fig. 8) [254].

#### 6.3.1 Nanozyme classification, catalytic mechanisms, and comparative advantages

Nanozymes are nanomaterials with intrinsic enzyme-like catalytic activity that can mimic the function of biological enzymes with superior physicochemical stability, tunable catalytic activity, and multi-enzyme-mimicking capacity. In the context of PD, nanozymes relevant to oxidative stress and neuroinflammation can be classified into five functional categories based on the reactions they catalyze. These include: (1) *superoxide dismutase (SOD)-mimetic nanozymes:* CeO_2_ nanoparticles, Mn_3_O_4_ nanocubes, and nitrogen-doped carbon dots catalyze the disproportionation of superoxide radicals (2O_2_•^-^ + 2H^+^ → H_2_O_2_ + O_2_). CeO_2_ achieves this via Ce^3+^ oxidation by O_2_•^-^, while Mn_3_O_4_ exploits the Mn^2+^/Mn^3+^ redox couple, (2) *Catalase (CAT)-mimetic nanozymes:* CeO_2_ (Ce^4^ + state) and Prussian blue nanoparticles (Fe^2^ +/Fe^3^ + cycle) catalyze H_2_O_2_ decomposition (2H_2_O_2_ → 2H_2_O + O_2_), preventing hydroxyl radical (•OH) generation via Fenton chemistry, (3) *Glutathione peroxidase (GPx)-mimetic nanozymes:* Selenium nanoparticles and MoS_2_ nanosheets catalyze hydroperoxide reduction coupled to GSH oxidation, as detailed in Section 6.3, 4) *Peroxidase (POD)-mimetic nanozymes:* Fe_3_O_4_ nanoparticles and Au nanoparticles catalyze H_2_O_2_-dependent substrate oxidation. While POD-mimetic activity can be exploited for diagnostic purposes, its pro-oxidant potential in the PD brain microenvironment warrants careful consideration, and (5) *Oxidase (OXD)-mimetic nanozymes:* V_2_O_5_ nanowires and MnO_2_ nanosheets can oxidize substrates using dissolved O_2_ as electron acceptors.

Compared with natural enzymes, nanozymes offer: (a) significantly superior thermal and pH stability (functional across pH 4-11 and temperatures up to 80°C vs. narrow physiological windows for natural enzymes); (b) resistance to proteolytic degradation; (c) multi-enzyme catalytic activity within a single nanomaterial (e.g., CeO_2_ exhibits SOD, CAT, and GPx-like activities simultaneously); (d) tunable catalytic activity through control of particle size, morphology, surface functionalization, and dopant incorporation; and (e) low manufacturing cost and scalable synthesis compared with recombinant enzyme production. Single-atom catalysts (SACs) represent an emerging class of nanozymes, in which individual metal atoms (Fe, Mn, Pt, and Co) are stabilized on nitrogen-doped carbon or metal-organic framework supports (M-N-C structures). SACs achieve near-100% atom utilization efficiency, thereby maximizing the catalytic activity per unit mass. Single-atom Fe-N-C SACs exhibit dual superoxide dismutase (SOD) and catalase (CAT) mimetic activities. Their intrinsic catalytic performance can be characterized using kinetic parameters such as *k*cat/*K*m; however, comparisons with conventional nanozymes must be interpreted within the conditions of the corresponding assay. In a rotenone-induced Parkinson's disease cellular model, treatment with single-atom Fe-N-C SACs at concentrations of 5–20 μg/mL reduced intracellular ROS by >70%, preserved mitochondrial membrane potential, and suppressed NF-κB activation. Under these specific experimental conditions, the effective dose of the Fe-N-C SACs was 10-50-fold lower than that of conventional CeO_2_ nanoparticles. This comparison reflects model- and dose-dependent cellular efficacy rather than a universal 10-50-fold superiority in intrinsic catalytic efficiency across different nanozyme systems or biological settings. The integration of nanozyme catalytic function within nanoplatforms designed for targeted brain delivery offers a compelling strategy to simultaneously address the oxidative stress, neuroinflammation, and mitochondrial dysfunction axes of PD pathogenesis.

### 6.4 Nanocarrier delivery of anti-inflammatory agents

Numerous anti-inflammatory compounds have been shown to have neuroprotective activity in PD; however, they have poor pharmacokinetics and CNS delivery. These drawbacks can be circumvented by using nanocarrier systems, which improve the solubility of drugs, protect therapeutic agents from degradation, and enable targeted delivery to the brain [244].

A great deal of research has been carried out on liposomal formulations because of their good biocompatibility and versatility in drug delivery systems. Prolonged circulation time and increased uptake in inflamed tissues are properties of liposome-encapsulated corticosteroids. Similarly, polymeric nanoparticles made of PLGA and its derivatives offer controlled drug release and long-lasting therapeutic action [220].

Nanoparticle delivery systems have significantly contributed to the use of natural active compounds with anti-inflammatory properties. Conventionally, curcumin, resveratrol, and quercetin are delivered to the body in a format that is poorly bioavailable; however, they possess strong anti-inflammatory and antioxidant activities. Stability, BBB penetration, and therapeutic efficacy in PD models are significantly enhanced by nanocarrier encapsulation [255].

Targeted delivery strategies further enhance these effects. Nanocarriers modified with transferrin, lactoferrin, peptides, or antibodies can specifically bind to receptors on the BBB and facilitate their uptake into damaged brain areas. The representative neuroprotective nanotherapeutic systems tested in PD models are summarized in Table 5 [263].

**Table 5:** Representative nanotherapeutic systems investigated for neuroprotection and immune modulation in PD models.

Nanotherapeutic System	Therapeutic Cargo	Primary Target	Neuroprotective Effects	References
Curcumin-loaded nanoparticles	Curcumin	Microglial activation, NF-κB signaling	Reduced neuroinflammation and oxidative stress	[246]
Resveratrol-loaded nanoparticles	Resveratrol	Oxidative stress and inflammatory pathways	Enhanced neuronal survival and mitochondrial protection	[256]
PLGA nanoparticles	GDNF, antioxidants, and anti-inflammatory agents	Dopaminergic neurons and glial cells	Sustained neuroprotection and improved motor function	[257]
Liposomal formulations	Corticosteroids, dopamine agonists	Neuroinflammation and dopamine deficiency	Enhanced brain delivery and reduced inflammation	[215]
Cerium oxide nanoparticles	Intrinsic antioxidant activity	ROS and mitochondrial dysfunction	Reduced oxidative damage and improved mitochondrial function	[258]
Selenium nanoparticles	Redox-active selenium compounds	Oxidative stress pathways	Antioxidant, anti-inflammatory, and neuroprotective effects	[259]
Exosome-based systems	siRNA, miRNA, proteins	Gene regulation and neuroimmune signaling	Efficient BBB penetration and targeted molecular delivery	[260]
Biomimetic nanoparticles	Anti-inflammatory agents, nucleic acids	Neuroinflammatory lesions	Enhanced targeting efficiency and immune evasion	[261]
LNPs	siRNA against SNCA	α-Synuclein pathology	Reduced α-synuclein accumulation and neuronal injury	[244]
Stimuli-responsive nanoplatforms	Antioxidants, RNA therapeutics, neuroprotective drugs	ROS-rich and inflamed microenvironments	On-demand drug release and improved therapeutic precision	[262]

### 6.5 RNA-based and gene-regulatory nanotherapeutics

Molecular neuroscience has shed new light on the pathogenesis of PD, revealing many molecular pathways involved in the disease, with the potential for RNA-based and gene-targeted therapies to treat PD in the future. However, nucleic acid therapeutics face significant delivery challenges owing to their low stability, degradation, and low cellular uptake. These challenges are critical and can be addressed using nanotechnology. Small interfering RNA (siRNAs), microRNAs (miRNAs), mRNA, antisense oligonucleotides, and gene-editing systems are examples of materials and tools that have been developed using nanocarriers. These strategies can be used to selectively regulate genes associated with inflammation, α-synuclein aggregation, mitochondrial dysfunction, and neuronal survival [200].

A specific area of interest is the silencing of SNCA expression, which decreases the production of α-synuclein. In experimental models, the targeted delivery of siRNAs against SNCA to the brain through LNPs/polymeric carriers has been shown to decrease the accumulation of the protein and improve neuronal survival. Likewise, the NF-κB and NLRP3 signaling pathways are important inflammatory pathways that have been shown to exert potent neuroprotective effects when modulated by RNA. Nanoparticle-mediated gene regulation opens new therapeutic windows for selectively targeting pathways involved in inflammation, accumulation of the pathological protein α-synuclein, and neuronal survival during disease progression (Fig. 8) [264].

Gene-editing technologies, such as CRISPR/Cas systems, have attracted much interest because of their ability to treat diseases over long periods. Nanoparticle-based delivery of gene-editing components is a safer option than that of viral vectors. They can be used to deliver components to specific genes associated with various diseases. Although still in its nascent stages of development, RNA-based nanotherapeutics are promising for advancing PD treatment [265].

### 6.6 Preclinical evidence supporting neuroprotection

There is increasing preclinical evidence validating the neuroprotective effects of nanotechnology-based approaches in PD. Ali et al. (2025) showed decreased neuroinflammation, oxidative stress, α-synuclein pathology, and dopaminergic neuron loss upon treatment with nanotherapeutic systems in toxin-induced and genetic animal models [266]. Representative examples include: (1) PLGA nanoparticles delivering GDNF (10 μg GDNF equivalent per injection, unilateral 6-OHDA rat model) produced a 45% improvement in rotarod performance at 4 weeks post-treatment compared with free GDNF controls, alongside significant preservation of striatal TH + fibre density; (2) curcumin PLGA nanoparticles (20 mg/kg i.p., MPTP mouse model) reduced striatal TNF-α and IL-1β levels by over 40% and attenuated loss of dopaminergic neurons in the SNpc; (3) CeO_2_ nanoparticles (0.5 mg/kg i.v., rotenone rat model) significantly reduced 8-hydroxy-2′-deoxyguanosine (8-OHdG) levels and preserved TH + cell count in the substantia nigra; and (4) LNP-encapsulated siRNA targeting SNCA (0.3 mg/kg i.v., AAV-SNCA overexpression mouse) achieved approximately 55% reduction in striatal α-synuclein protein levels at 3 weeks, with corresponding improvements in pole test performance.

Dopamine agonists, antioxidants, and anti-inflammatory compounds carried by lipid-based nanoparticles have been found to enhance motor function and neuronal survival. Neurotrophic factors or gene-regulatory molecules are potential therapeutic agents that have been shown to have prolonged activity when delivered using polymeric nanoparticles and are more neuroprotective than other agents. Cerium oxide nanoparticles and other redox-active materials have been shown to have exceptional antioxidant properties and the ability to maintain mitochondrial function against oxidative stress.

Promising new strategies have been developed to deliver therapeutic RNA molecules across the BBB using exosome-based delivery systems with low immunogenicity. Targeting efficiency is further enhanced with biomimetic nanoparticles that use endogenous routes of immune traffic. Altogether, these strategies provide proof of principle of the capacity of nanoplatforms to act simultaneously on the four processes of inflammation, oxidative stress, mitochondrial dysfunction, and gene expression, contributing to neuronal survival and functional recovery in the brain [267].

Although translation to the clinic is not imminent, there is a strong body of evidence supporting the neuroprotective effects of nanomedicine in PD. The ongoing development of nanotechnology, targeting, manufacturing standardization, and safety optimization is anticipated to accelerate the development of clinically viable neuroprotective agents. Nanotherapeutic platforms, along with their cargos, targets, and neuroprotective outcomes, are summarized in Table 5 [268].

## 7 Nanoplatforms for disease modification and α-synuclein pathology

However, although neuroprotection has been an important therapeutic goal in PD, more focus has been directed towards disease-modifying therapies that can target the mechanisms underlying disease progression. These include the aggregation and propagation of α-synuclein, which are key pathological processes. The formation of toxic α-synuclein oligomers and fibrils leads to mitochondrial dysfunction, neuroinflammation, lysosomal dysfunction, synaptic dysfunction, and progressive neuronal death. Therefore, therapeutic approaches targeting the reduction of α-synuclein burden or pathological spreading have become key targets in PD research [194].

Nanotechnology offers unprecedented opportunities to modify diseases and take advantage of its ability to deliver drugs to the brain, enhance intracellular targeting, regulate genes, and integrate multiple therapeutic functions on a single platform. In recent years, nanoplatforms have been shown to facilitate the clearance of α-synuclein, prevent its aggregation, restore degradative processes, control the expression of disease-associated genes, and integrate diagnostic and therapeutic functions for precision medicine [269].

### 7.1 Nanotechnology approaches for α-synuclein clearance

**Fig. 9:**
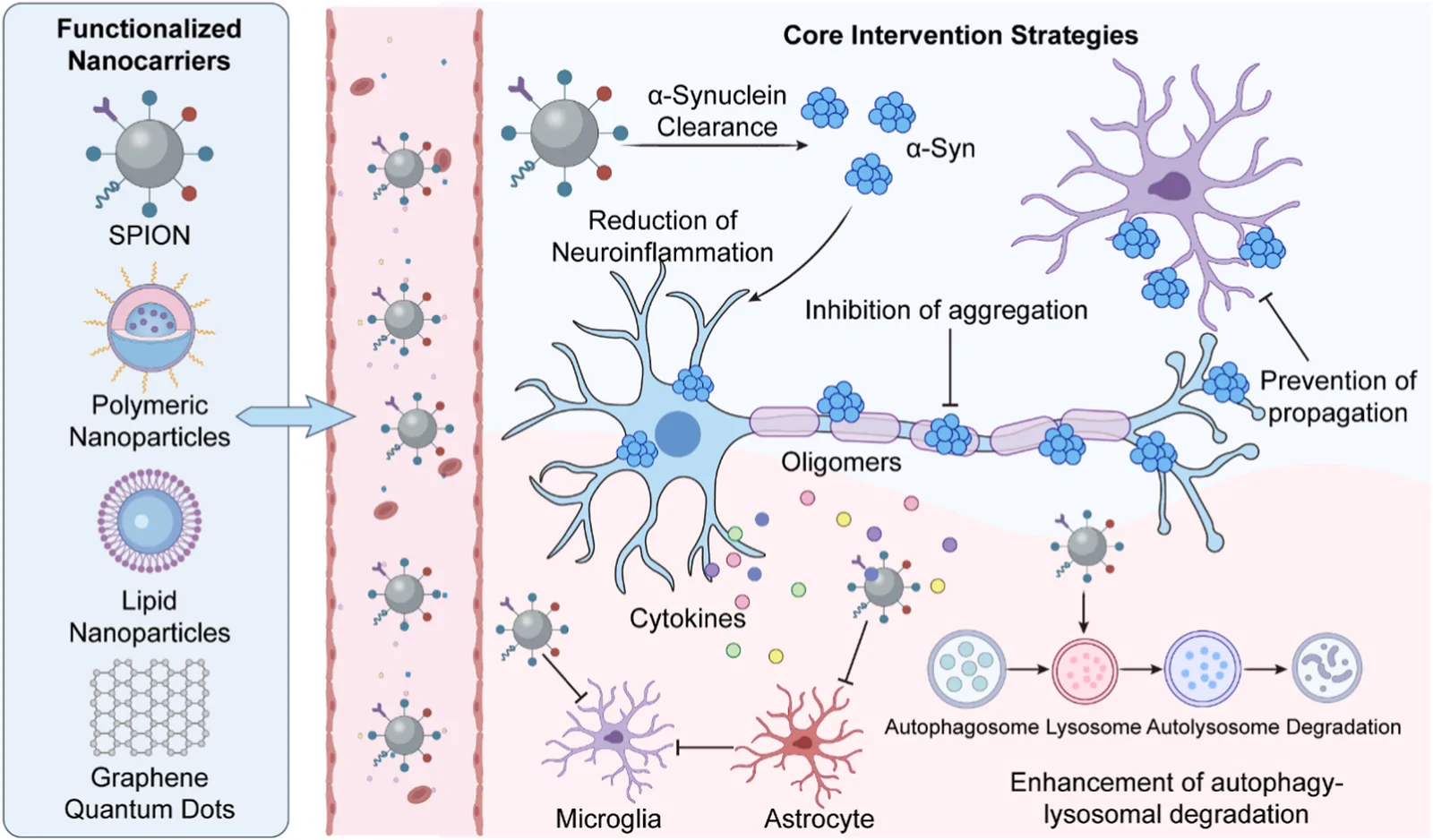
Nanotechnology-based strategies targeting α-synuclein pathology in PD Nanoplatforms promote α-synuclein clearance, inhibit aggregation and fibril formation, prevent cell-to-cell propagation, enhance autophagy–lysosomal degradation, reduce neuroinflammation, and improve therapeutic delivery across the BBB [270]. Created by the authors using Adobe Illustrator.

Pathological α-synuclein accumulation is caused by an imbalance between protein synthesis, aggregation, and degradation. Thus, improving the clearance of toxic species of α-synuclein is an important strategy for disease modification in PD. Nanotechnology-based strategies can facilitate the clearance of α-synuclein by enhancing its uptake, lysosomal degradation, and immune clearance, thereby reducing the pathological protein load in affected brain areas (Fig. 9) [271].

Molecular chaperones, proteins involved in the regulation of protein folding, proteostasis proteins, and compounds that promote protein degradation have been used to modify the activity of nanocarriers, enabling them to fold proteins properly and boost the clearance of misfolded proteins. Therapeutic cargo is also encapsulated within the nanoparticles, shielding it from enzymatic degradation and aiding in crossing the BBB to achieve effective delivery to diseased brain regions, as reported by Sharma et al. (2023) [272].

The use of nanotechnology is also advantageous for immunotherapeutic approaches. Nanoparticle-based vaccines are a new class of vaccines that improve antigen presentation and promote antibody production against pathological conformations of the α-synuclein protein. Likewise, nanocarriers can help cross the BBB, overcoming a major limitation of conventional immunotherapy that involves carrying monoclonal antibodies. These methods maximize aggregate clearance but reduce systemic toxicity [273].

The advantage of exosome-based delivery systems is their natural capacity to penetrate biological barriers and communicate between cells. Engineered exosomes loaded with antibodies, therapeutic proteins, or nucleic acids have demonstrated efficacy in experimental models of PD, decreasing α-synuclein accumulation and enhancing neuronal survival [274].

### 7.2 Inhibition of aggregation and cell-to-cell propagation

Soluble α-Synuclein has been shown to be converted into toxic oligomers and fibrils, which play a key role in the pathogenesis of PD. Furthermore, pathological α-synucleins can spread along interlinked neural networks by prion-like transmission from neuron to neuron and from glial cells to other glial cells, which could help trigger the disease process. Beyond their clearance-promoting properties, nanoplatforms can directly block α-synuclein aggregation and disrupt cell-to-cell propagation mechanisms involved in the spread of disease (Fig. 9) [275].

Several types of nanomaterials, including gold nanoparticles, graphene-based nanomaterials, polymeric nanoparticles, and peptide-functionalized systems, have been shown to inhibit fibril formation or disrupt pre-existing aggregates. The selectivity towards pathological protein conformations can be further improved by surface modification, and interactions with normal cellular proteins can be minimized [276].

A potentially useful approach is to block intercellular transmission. Neighboring cells may take up less extracellular α-synuclein if it binds to a nanocarrier containing aggregation inhibitors, receptor-blocking molecules, or neutralizing antibodies. These interventions can have beneficial effects on the spread of pathology throughout the nervous system [277].

Recent research has focused on nanoparticles that can scavenge extracellular α-synuclein aggregates, known as molecular scavengers. These systems help limit the availability of aggregates for propagation and dampen inflammatory responses to the accumulation of extracellular proteins [278].

### 7.3 Nanoplatform-assisted autophagy and lysosomal restoration

Dysfunction of the autophagic and lysosomal pathways has emerged as a key factor in PD. Dysfunctional autophagy and lysosomal breakdown are key factors in the accumulation of α-synuclein in PD. These pathways also function in normal cells to eliminate damaged proteins and organelles, thereby maintaining proteostasis under normal physiological conditions. Failure of degradative systems leads to the formation of aggregates and higher neuronal sensitivity [279].

Another key approach to decreasing α-Syn accumulation and toxicity is the restoration of autophagic and lysosomal pathways (Fig. 9). Intracellular degradation mechanisms can be enhanced by loading nanoplatforms with agents that induce autophagy, such as rapamycin, trehalose, and related compounds. Controlled release is another method for enhancing effectiveness by providing sustained intracellular drug concentration [280].

Targeting the regulators of lysosomal biogenesis and autophagy using a gene-based approach has also shown promise. Restoring lysosomal function using nanocarriers that deliver nucleic acids to regulate transcription factors in the degradation pathway can restore aggregate clearance. Similarly, enzyme-containing nanoparticles can be used to repair lysosomes with defects resulting from mutations (e.g., GBA1 deficiency) [281].

Since there are strong connections between autophagy, mitochondrial quality control, and neuroinflammation, restoring naturally occurring pathways may also improve several aspects of PD pathology. Therefore, targeting pathways associated with cellular clearance mechanisms (CCM) as a disease-modifying approach using nanoplatforms is a promising strategy currently under investigation [279].

### 7.4 Gene silencing and genome editing approaches

The identification of PD-associated genes has provided opportunities for specific molecular interventions. Of these, SNCA, which encodes α-synuclein, is the most important PD-modifying therapeutic target. However, the efficient delivery of nucleic acid therapeutics to the CNS remains a significant challenge [282].

**Fig. 10:**
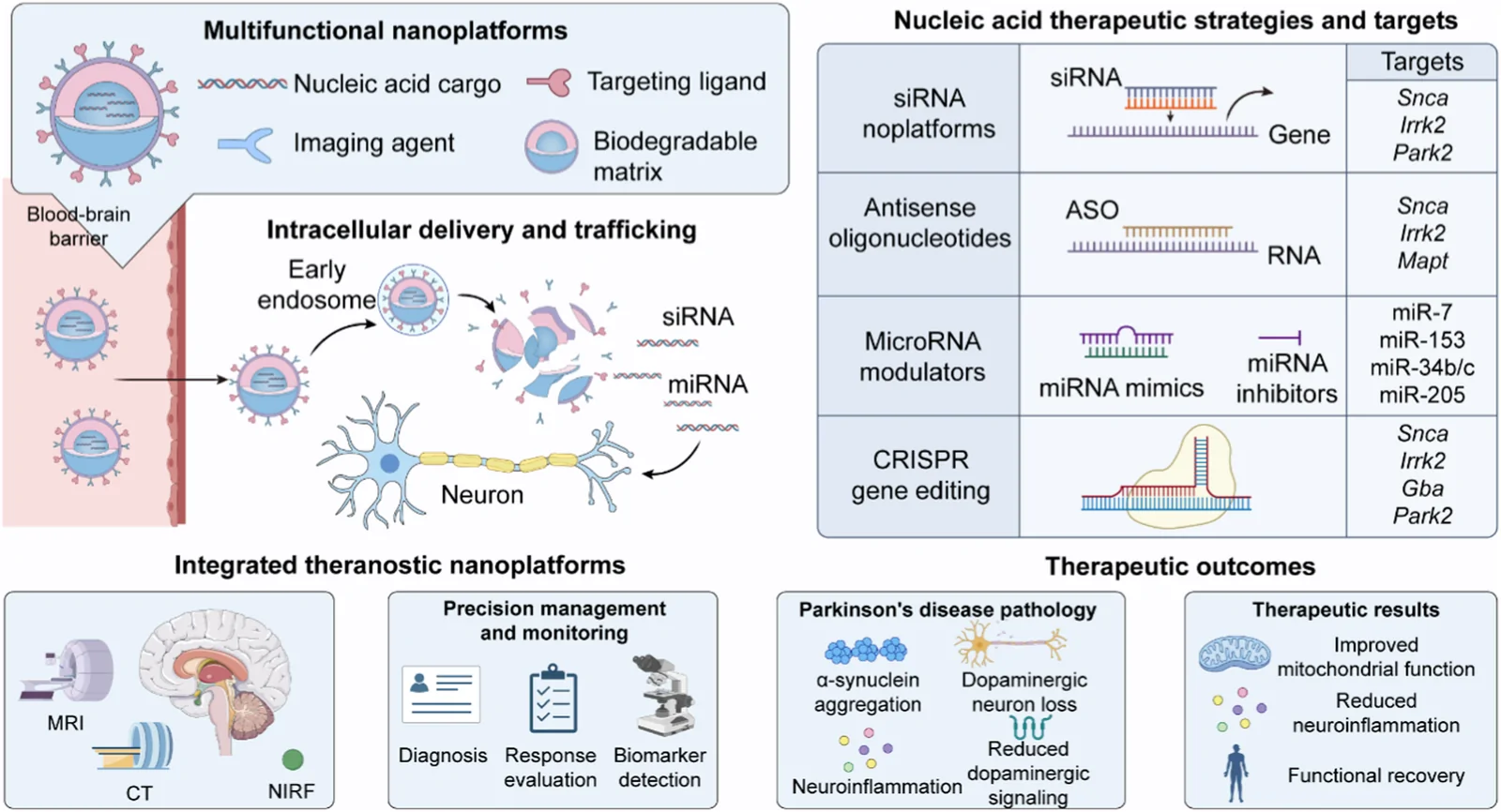
Gene, RNA, and theranostic nanoplatforms for disease modification in PD. Multifunctional nanocarriers enable the delivery of siRNAs, antisense oligonucleotides, microRNA modulators, CRISPR/Cas gene-editing systems, and imaging agents for precision therapy, disease monitoring, and personalized treatment strategies [283]. Created by the authors using Adobe Illustrator.

siRNAs, antisense oligonucleotides (ASOs), microRNA modulators, and gene-editing systems are potential tools for molecular disease modification using nanocarriers (Fig. 10). Preclinical applications of gene-silencing therapeutics that target SNCA expression include LNPs, polymeric nanocarriers, dendrimers, and exosome-based systems. A consistently reduced rate of α-synuclein synthesis decreases protein levels and increases neuronal survival. Other targets include LRRK2, GBA1, PINK1, PRKN, MAPT, and inflammatory signaling regulators, as well as SNCA. Nanocarrier-mediated gene modulation enables the selective regulation of these pathways with minimal systemic toxicity and off-target effects [264].

Genome editing tools, such as CRISPR/Cas systems, have therapeutic potential. Gene-editing machinery delivery via nanoparticles offers several advantages over viral vectors, including low immunogenicity, safety, and design flexibility. Although there are still substantial hurdles to overcome in terms of the efficiency, specificity, and long-term safety of these genomic editing techniques, they offer a promising approach for making permanent changes [284].

### 7.5 Multifunctional disease-modifying nanoplatforms

As several factors are involved in PD, it is unlikely that actions taken in a single direction will produce significant changes. Therefore, multifunctional nanoplatforms capable of targeting multiple disease pathways have received increasing attention.

Multiple therapeutic modalities were combined into one system. For instance, an anti-inflammatory compound, an antioxidant agent, and a gene-silencing agent against α-synuclein expression could be co-delivered by a nanoparticle. These integrated interventions have synergistic effects by targeting different interconnected mechanisms, such as protein aggregation, oxidative stress, neuroinflammation, and mitochondrial dysfunction in the brain [278].

In the last few years, multifunctional nanoplatforms have become increasingly capable of incorporating several functions in the same therapeutic system, such as protein targeting, gene regulation, and anti-inflammatory and antioxidant effects (Fig. 10). Selective targeting of ligands can aid in their delivery to neurons, microglia, astrocytes, and the pathological areas of the brain. Stimuli-responsive components can also allow for site-specific release depending on the microenvironmental conditions of the disease and the presence of oxidative stress or inflammatory enzymes. Such advanced designs achieve maximum therapeutic efficacy and result in a minimum systemically absorbed dose, thus being highly compatible with new precision nanomedicine approaches [285]. Importantly, the following safety considerations are derived primarily from mechanistic reasoning and evidence obtained in preclinical studies. At present, multifunctional nanozyme-integrated nanoplatforms have not yet advanced sufficiently to provide a substantive body of published human safety data. To date, no published clinical studies have reported adverse events specifically attributable to multifunctional nanozyme-integrated systems. However, the absence of reported adverse events should not be interpreted as evidence of established clinical safety, but rather as a reflection of the limited clinical evaluation of these emerging platforms. Accordingly, the potential risks discussed below should be interpreted as anticipated translational concerns that require systematic experimental and clinical validation, rather than as confirmed adverse outcomes in patients. Despite the promise of multifunctional nanoplatforms, the simultaneous targeting of multiple molecular pathways introduces risks that require careful consideration. First, broad multi-target engagement increases the probability of off-target effects in peripheral immune organs (liver, spleen, and lymph nodes), potentially causing hepatotoxicity, cytokine dysregulation, or unintended systemic immunosuppression. Second, co-suppression of NF-κB, NLRP3, and pro-inflammatory cytokines while simultaneously promoting Treg expansion may impair physiological immune surveillance, increasing susceptibility to infection or tumor immune evasion. Third, the engineering complexity of nanoplatforms incorporating ≥3 functional components (e.g., targeting ligands, gene silencing, anti-inflammatory drugs, and imaging agents) introduces substantial batch-to-batch variability in size distribution, surface functionalization, and drug loading ratios, significantly complicating GMP-compliant manufacturing and regulatory characterization. These considerations underscore the need for rigorous mechanistic safety studies, dose–response profiling, and target prioritization when designing multifunctional nanoplatforms for clinical translation.

### 7.6 Theranostic platforms for simultaneous diagnosis and therapy

One of the major advancements in nanomedicine is the development of theranostic systems that combine diagnostic and therapeutic properties into a single system. These systems allow real-time monitoring of disease progression, response to treatment, and optimization of personalized treatment strategies [286].

The combination of molecular imaging, biomarker monitoring, and targeted therapy has led to rapid progress in the development of theranostic nanoplatforms for the precision management of PD (Fig. 10). Nanoparticles can be used to simultaneously transport therapeutic cargo and imaging agents at the same time, enabling the visualization of pathological processes for treatment. For instance, magnetic nanoparticles can be used in magnetic resonance imaging and to carry a neuroprotective/anti-aggregative compound to the target site [200].

Likewise, nanoplatforms that can be used for fluorescence, photoacoustic, and positron emission tomography (PET) allow for the sensitive detection of pathological changes and therapeutic responses. Several systems have been designed to detect α-synuclein aggregates and provide aggregation inhibitors, gene regulators, and neuroprotective agents [287].

Improved BBB penetration and tissue specificity are achieved with exosome-based and biomimetic nanoplatforms, which further enhance theranostic capabilities. With the rapid development of molecular diagnostics, imaging, and nanomaterial engineering, theranostic nanoplatforms have become important tools for individualized disease-modifying interventions in patients with PD [267].

## 8 Nanoplatform-assisted neural repair and regeneration

**Fig. 11:**
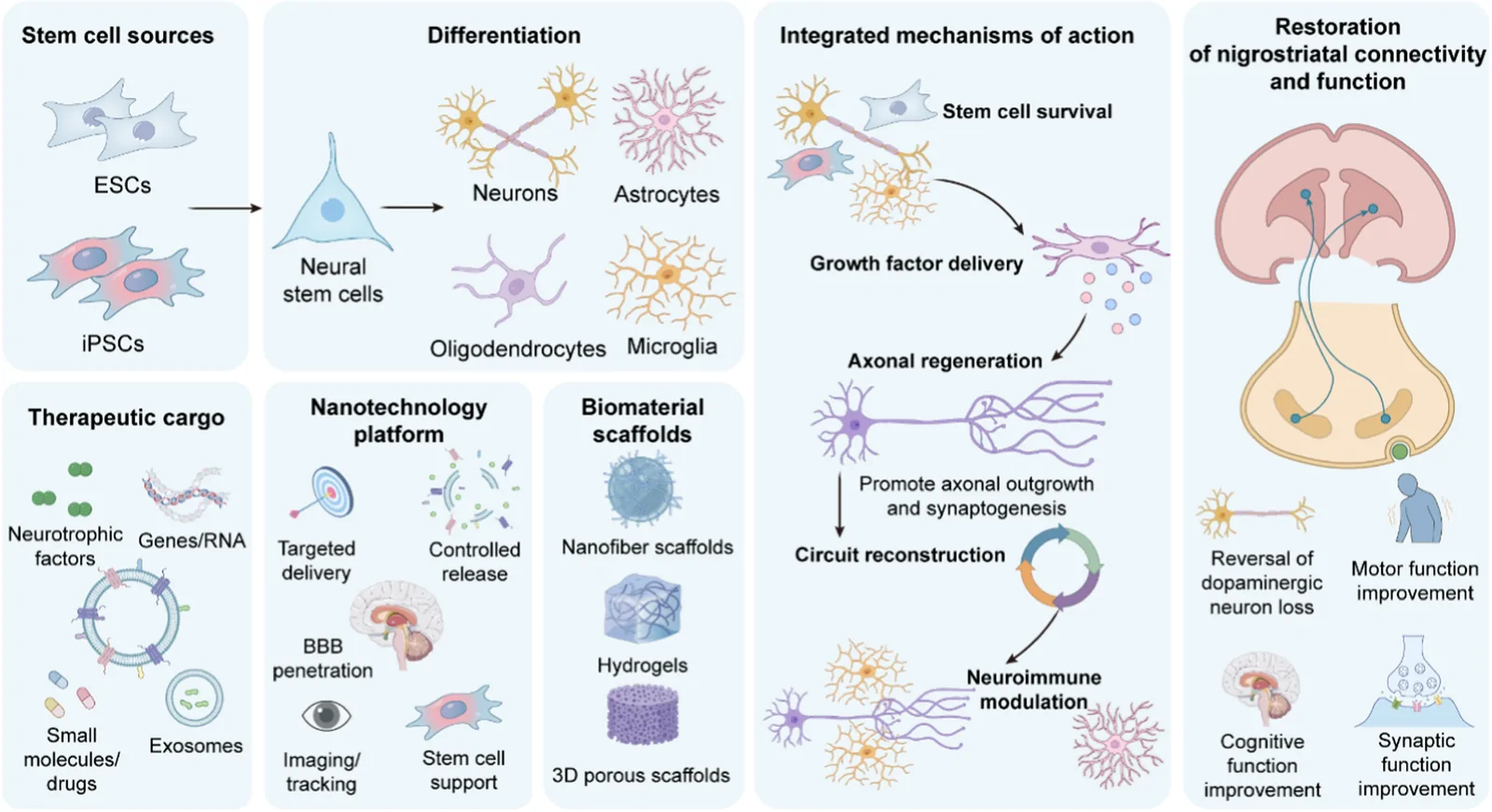
Nanotechnology-enabled neural repair and regeneration in PD. The integration of stem cell therapies, neurotrophic factor delivery, biomaterial scaffolds, axonal regeneration, circuit reconstruction, and neuroimmune modulation restores nigrostriatal connectivity and improves functional recovery [288]. Created by the authors using Adobe Illustrator.

Although the ultimate therapeutic goals of PD include neuroprotection and disease modification, such approaches are unlikely to fully correct the loss of neurons and restore disrupted neural circuits. Degeneration of dopaminergic neurons in the SNpc and subsequent disconnection of the nigrostriatal pathway cause chronic motor and non-motor deficits. Therefore, there has been an increasing trend in employing regenerative approaches that aim to replace damaged cells, restore neural network connectivity, and reconnect functions. With the advent of nanotechnology, there are many exciting possibilities for improving neural repair through the targeted delivery of regenerative factors, promotion of the survival of stem cells, functional engineering of biomimetic scaffolds, and modulation of the neuroimmune response that regulates tissue regeneration (Fig. 11) [289,290].

### 8.1 Neurorestorative concepts in PD

Neurorestoration is the process of restoring neural function via mechanisms that facilitate the repair, replacement, or regeneration of damaged tissues. Neurorestorative methods are directed towards reestablishing neuronal circuits and restoring physiological functions, whereas symptomatic methods are designed to compensate for low levels of dopamine. These strategies targeted at PD are aimed at preserving the remaining dopaminergic neurons, inducing a repair process, replacing lost dopaminergic neurons, and strengthening neural plasticity [291].

Neurogenesis, axonal sprouting, and synaptic remodeling are important mechanisms of recovery in the adult brain, but are often reduced by chronic neuroinflammation, oxidative stress, alpha-synuclein pathology, and mitochondrial dysfunction. Thus, to regenerate damaged nerves, it is necessary to change the hostile microenvironment that impedes the repair process and stimulates regeneration [292].

In this regard, nanotechnology has several advantages. Nanoplatforms are used to deliver neurorestorative molecules, improve cellular survival, and generate microenvironments conducive to regeneration. Furthermore, engineered nanomaterials can directly interact with neural cells and ECM components and aid in repairing the nervous system at various levels [293].

### 8.2 Nanomaterial-enhanced stem cell therapies

The use of stem cell-based therapies is one of the most promising regenerative therapies for PD. Neuronal lineages can be generated from embryonic stem cells (ESCs), induced pluripotent stem cells (iPSCs), neural stem cells (NSCs), and mesenchymal stem cells (MSCs), which may replace lost dopaminergic neurons. However, in clinical applications, it is difficult to achieve a high survival rate of transplanted cells, where the cells do not integrate or differentiate well, and the inflammatory microenvironment can attack the transplanted cells [294].

To overcome these drawbacks, nanotechnology has recently shown great potential for improving stem cell viability, differentiation, and long-term engraftment in the diseased microenvironment of the brain (Fig. 11). Nanoparticles can be used to transport growth factors, transcription factors, and genetic regulators to guide the maturation of stem cells into dopaminergic phenotypes. Magnetic nanoparticles have also been used to target and deliver stem cells to the brain's target areas using an external magnetic field [295].

Physical and biochemical cues control stem cell behavior and integration, with nanostructured surfaces and biomimetic materials providing these cues. In addition, it is possible to label cells with nanoparticles, which will allow noninvasive tracking of cells using MRI and other imaging techniques during therapy and observation of their survival, migration, and therapeutic effects in real time [296].

### 8.3 Neurotrophic factor delivery systems

Neurotrophic factors play critical roles in neuronal survival, differentiation, growth, and plasticity. Several molecules have been identified to show significant regeneration in experimental PD models, including glial cell line-derived neurotrophic factor (GDNF), brain-derived neurotrophic factor (BDNF), nerve growth factor (NGF), and neurturin. However, these peptides have limitations in their clinical application due to the barrier effect of the BBB, degradation, and short half-lives [297].

Nanotechnology is a potential solution to these problems. Neurotrophic factors can be protected from degradation and released in a sustained manner over a longer period by encapsulating them into liposomes, polymeric nanoparticles, hydrogels, and exosome-based carriers. To increase therapeutic efficacy, these molecules can be delivered using targeted delivery systems that increase their concentration in the affected brain regions while decreasing systemic exposure [298].

Moreover, gene delivery nanoplatforms can upregulate the regeneration-supportive expression of neurotrophic factors in host tissues for a long period. In addition, the delivery of neurotrophic factors in combination with anti-inflammatory and antioxidant treatments could enhance the results by providing an environment that is more favorable for the survival of neurons and circuit reconstruction [299].

### 8.4 Biomaterial scaffolds for neural regeneration

The ECM is a key determinant of neuronal growth, extension, and organization. These functions are mimicked by biomaterial scaffolds that offer structural support and biochemical cues to promote regeneration.

Collagen, chitosan, hyaluronic acid, graphene, and biodegradable polymers have been used to create nanostructured scaffolds that are promising for neural tissue engineering. These materials can be modified into 3-dimensional structures that promote cellular attachment, migration, differentiation, and survival, resembling native neural microenvironments [300].

Multifunctional biomaterial scaffolds can provide structure, serve as reservoirs for regenerative factors, and even act as delivery platforms for stem cells for neural tissue reconstruction (Fig. 11). Such scaffolds facilitate better retention of transplanted cells and their integration into host neural circuits in PD. Graphene and carbon nanotube-based composites could be used to improve the electrical signaling of neurons and communication between them, which will help to restore functional connectivity [301].

### 8.5 Synaptic plasticity and circuit reconstruction

Recovery of function in PD requires not only the survival of neurons but also the restoration of the connectivity of neural networks. Thus, the ability of synapses to vary their strength, axons to regenerate, and circuits to reconstruct are essential features of successful regenerative therapy. Nanotechnology can impact these processes in several complementary ways [200].

Neurotrophic factors are delivered, causing neurite extension, axonal growth, and synaptogenesis, while nanostructured scaffolds physically guide neurite outgrowth. Developmental signaling molecules can be further delivered to synapses by nanocarriers to enhance and strengthen functional connectivity. Owing to their ability to directly regulate neuronal activity, electroactive nanomaterials have gained increasing interest. Conductive scaffolds improve electrical communication between neurons, and nanocomposites may aid in integrating newly formed neural networks. These strategies are particularly pertinent to the reconstructed and disrupted nigrostriatal circuitry underlying PD [302].

Emerging evidence suggests that exosome-based systems play a role in the repair of synapses by delivering proteins, lipids, and regulatory RNAs involved in neuronal communication and plasticity. These mechanisms enable neuronal replacement and the restoration of complex neural networks necessary for functional recovery, which is supported by nanotechnology [303].

### 8.6 Neuroimmune regulation during neural repair

A key factor in successful regeneration is neuroimmune signaling. The tissue repair process is hampered by excessive inflammation; however, controlled immune responses are needed for the clearance of debris, angiogenesis, and tissue remodeling to restore homeostasis. Thus, regenerative treatments should selectively target inflammation and maintain the ability of the immune system to act positively [304].

Microglia and astrocytes play a central role in this process. Microglia in homeostasis release trophic factors for regeneration and promote synaptic remodeling, whereas chronically activated microglia are neurogenic, and neuronal survival is impaired. Similarly, astrocytes can promote repair by providing trophic support or inhibiting regeneration through a persistent reactive phenotype [305].

Such modulation of these processes is possible using nanoplatforms. Microenvironments conducive to regeneration can be created by the targeted delivery of anti-inflammatory agents, cytokine regulators, neurotrophic molecules, and immunomodulatory nucleic acids. Biomimetic nanoparticles and exosome-based systems can target endogenous repair pathways by improving communication between neurons, glial cells, and regenerative cell populations [306].

Nanoplatform-based interventions have a holistic approach, promoting neuronal regeneration, restoring immune homeostasis, and reconstructing nigrostriatal circuitry, thus enabling long-term functional recovery in PD (Fig. 11) [265].

## 9 Translational challenges and clinical prospects

**Fig. 12:**
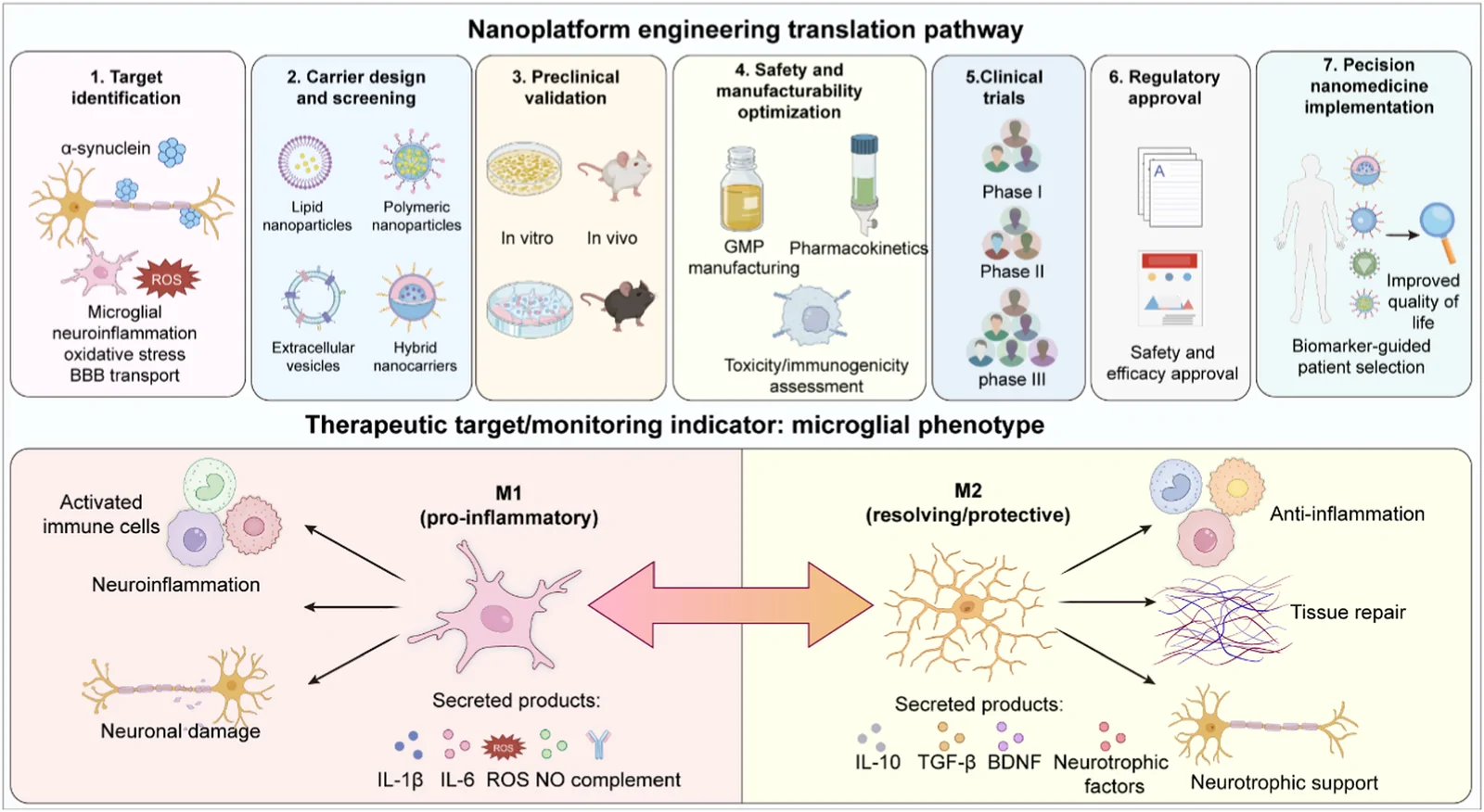
Translational roadmap for neuroimmune nanomedicine in PD progression from target identification and nanoplatform engineering through preclinical validation, safety optimization, clinical trials, regulatory approval, and implementation of precision nanomedicine approaches for PD [307]. Created by the authors using Adobe Illustrator.

Although great progress has been made in elucidating the neuroimmune mechanisms of PD and the emergence of highly developed nanotherapeutic platforms, clinical translation remains a challenge. While many nanomedicine interventions have been shown to be effective in cellular and animal models, only a few have been promoted for clinical testing in humans. Scientific, technological, manufacturing, regulatory, and clinical hurdles must be overcome to move from proof of concept to approved therapies. To maximize the potential of nanotechnology-based neuroimmune therapeutics and translate them into clinical use, these challenges must be addressed (Fig. 12) [265,290].

### 9.1 Safety and long-term biocompatibility

Safety is among the most significant features to be considered in developing nanomedicines for neurodegenerative diseases. Owing to their unique physicochemical properties, the biological interactions of nanoparticles are complicated and difficult to predict, as they differ from the interactions of conventional therapeutics. Toxicity profiles vary greatly depending on factors such as particle size, shape, surface charge, chemical composition, and degradation products [308].

One of the primary issues is the gradual accumulation of these substances in the body tissues over time. They can remain in organs, including the liver, spleen, kidneys, and brain, for a long time and cause chronic toxicity or unexpected biological effects. The potential for long-term *in vivo* persistence and induction of oxidative stress has led to interest in inorganic nanomaterials, especially metallic nanoparticles [309].

Immunogenicity is another challenge, and numerous nanocarriers have been engineered to avoid detection by the immune system. However, repeated dosing can cause immune responses that impact biodistribution, therapeutic effects, or create more adverse events. Therefore, it is crucial to evaluate these nanocarriers in detail for biodistribution, neurotoxicity, immunotoxicity, and long-term biocompatibility prior to their clinical use. The development of biodegradable materials, biomimetic systems, and naturally derived nanocarriers has become a priority, with an increased focus on enhancing safety profiles [310].

### 9.2 Pharmacokinetic and biodistribution considerations

One of the key challenges in nanomedicine is the efficient and reproducible delivery of drugs to the desired brain areas. The pharmacokinetics of nanoparticles can be affected by various factors, such as their size, surface properties, protein corona, route of administration, and interactions with biological barriers [311].

Although numerous nanocarriers can cross the BBB more efficiently than traditional therapeutics, they suffer from poor delivery efficiency, which may be due to low translocation rates. Shaw et al. (2025) reported that many nanoparticles may be taken up by the reticuloendothelial system and thus be retained in the liver and spleen, but not the CNS [312].

Multiple brain regions and a variety of cell populations are implicated in the pathology, and these challenges are especially relevant in PD. Therefore, therapies that selectively target dopaminergic neurons and/or microglia, astrocytes, or neurovascular components are required for effective treatment. Therefore, targeting ligands, administration, and controlled release routes continue to be a large field of investigation. In addition, advanced imaging technologies and theranostic nanoplatforms enable real-time monitoring of biodistribution and therapeutic responses [313]. A critical quantitative constraint that must be explicitly acknowledged is BBB penetration efficiency. As noted in Section 5.2, even optimized targeted nanoparticles typically achieve brain parenchymal uptake of less than 0.1–1% of the injected dose. In practical terms, this means that for a hypothetical therapeutic nanoparticle administered at 10 mg/kg, fewer than 0.1 mg/kg reaches the target brain tissue—raising significant questions about achieving therapeutic drug concentrations in the SNpc while remaining below systemic toxicity thresholds. Overcoming this fundamental limitation will likely require a paradigm shift toward combination approaches: focused ultrasound-mediated transient BBB opening, intranasal nose-to-brain delivery bypassing the BBB entirely, or implantable sustained-release depot systems that provide local therapeutic concentrations.

### 9.3 Manufacturing and regulatory challenges

Laboratory research must be translated to commercial production, and scalable, reproducible, and cost-effective manufacturing processes are required. Numerous nanoplatforms appear to perform well for experimental purposes but have significant problems with scaling up [314].

Batch-to-batch variability is a significant problem, as small changes in size, surface chemistry, drug loading, and release rate may have a significant impact on the biological response and therapeutic activity. Hence, ensuring consistency in the manufacturing process is crucial for regulatory approval and clinical implementation [315].

To translate the process of target identification and engineering of nanoplatforms into clinical approval, the manufacturing, safety, and regulatory processes must be optimized in tandem (Fig. 12). Nanotherapeutics offer a combination of drugs, biologics, medical devices, and advanced material properties, which pose unique regulatory challenges in contrast to traditional drugs. Physicochemical properties, safety profiles, biological interactions, and manufacturing quality must be comprehensively characterized for approval by regulatory agencies.

Moreover, multifunctional nanoplatforms with targeting ligands, imaging agents, and multiple therapeutic payloads may require more consideration for clinical applications. It is crucial for the translation of nanomedicine into clinical use, with the harmonization of international regulatory requirements and development of nanomedicine-specific frameworks [316,317]. Specifically, GMP-compliant manufacturing of nanoplatforms faces several technical challenges that are not encountered with conventional small-molecule drugs. For LNPs, the ionizable lipid pKa, lipid molar ratios, and microfluidic mixing parameters must be tightly controlled to maintain a consistent particle size distribution (polydispersity index ≤0.2) and nucleic acid encapsulation efficiency (>85%), as deviations directly impair gene silencing efficacy and safety. Polymeric nanoparticles face batch-to-batch variability in surface functionalization density, which affects targeting efficiency and immunogenicity. Cold-chain storage requirements (typically −80°C for mRNA-LNPs) complicate global distribution. Sterile filtration through 0.22 μm membranes, standard for conventional injectables, is incompatible with nanoparticles >200 nm, necessitating alternative sterilization approaches (e.g., gamma irradiation, aseptic processing) that may alter nanoparticle integrity. The comprehensive physicochemical characterization packages required by the FDA/EMA for nanomedicine approval (particle size, zeta potential, morphology, drug loading, release kinetics, sterility, endotoxin content, and immunogenicity testing) are significantly more resource-intensive than those for small molecules, contributing to higher development costs and longer regulatory timelines.

The predictive value of preclinical nanomedicine data is further constrained by the inherent limitations of the available PD animal models. Toxin-induced models (MPTP, 6-OHDA, and rotenone) produce rapid and severe nigrostriatal degeneration within days to weeks, enabling the efficient screening of neuroprotective nanoplatforms; however, they do not recapitulate the progressive α-synuclein aggregation, Lewy body formation, or gut-to-brain pathology propagation that characterizes human PD. Genetic models (SNCA-overexpressing, LRRK2-G2019S knock-in, and GBA1-knockout mice) better reflect specific molecular aspects of human PD pathogenesis but exhibit slow and incomplete dopaminergic degeneration, making it difficult to evaluate meaningful neuroprotective endpoints within standard study timescales. Crucially, neither model type fully recapitulates the complex human neuroimmune landscape, including the ratio of M1/M2 microglia, degree of T cell infiltration, BBB permeability, and systemic inflammatory milieu, which ultimately determines nanomedicine efficacy and safety in patients with PD. These limitations underscore the need for complementary human-relevant experimental systems that more accurately reproduce PD-associated neuronal, neuroimmune, and neurovascular features. As discussed in Section 10.6, human midbrain organoids and BBB-on-a-chip microfluidic systems provide potential solutions by enabling the evaluation of patient-relevant disease phenotypes, nanoparticle transport, therapeutic efficacy, and toxicity under more physiologically representative conditions. Nevertheless, rigorous validation against human post-mortem and biomarker data remains essential before clinical translation.

### 9.4 Precision nanomedicine and patient stratification

There is a significant clinical and biological heterogeneity in PD. There is significant inter-patient variability in genetic, disease course, inflammatory, α-synuclein pathological, and therapeutic responsiveness variables. This variability may be one reason why uniform treatment strategies have failed to be successful and highlights the need for precision-based medicine, as reported by Landolfi et al. (2025) [318].

The use of advances in genomics, transcriptomics, proteomics, metabolomics, and biomarker discovery has increasingly led to the stratification of patients based on the underlying disease mechanisms. The identification of neuroimmune signatures could help identify patients who are most likely to benefit from specific therapeutic interventions [319].

The trend of precision nanomedicine increasingly includes biomarker-based patient selection, molecular diagnostics, and personalized therapeutic strategies (Fig. 12). Nanoplatforms are particularly well-suited for such applications, as they can be designed to deliver tailored therapeutic combinations based on the patient's pathological profile. Nanotherapies, including immunomodulatory drugs, may be beneficial in cases with a significant neuroinflammatory phenotype. Simultaneously, gene-targeted therapies may be more effective in cases with a significant genetic component. The future integration of molecular diagnostics, artificial intelligence, and personalized nanomedicine is likely to make therapy more efficient and prevent unnecessary treatment [320].

### 9.5 Clinical trials and emerging therapeutic candidates

While most nanomedicine-based methods for PD are still in the preclinical stage, interest in the disease is growing in clinical trials. Xu et al. (2024) reported that a few types of nanoparticles used for neurological or systemic diseases have been found to be safe and suitable for use in neurodegenerative diseases [268].

Much of the current therapeutic focus is on enhancing the delivery of current therapies, such as dopamine replacement agents, neuroprotective compounds, and gene-based therapeutics. LNPs have successfully delivered nucleic acids and are currently being investigated for CNS applications. Likewise, the natural biocompatibility and penetrating capabilities of the BBB have led to extensive interest in exosome-based systems [321].

As new and emerging α-synuclein immunotherapies, RNA therapeutics, and genome-editing techniques emerge, they will continue to broaden the potential applications of nanotechnology-based therapies. Definitive disease-modifying nanotherapies have not yet been introduced into clinical use, but several are moving forward to advanced preclinical and clinical development. Table 6 highlights the main nanomedicine platforms in the translational development process of PD [265].

**Table 6:** Clinical and translational status of nanomedicine-based interventions for PD.

Therapeutic Platform	Primary Therapeutic Target	Mechanism of Action	Development Status	References
Liposomal dopamine formulations	Dopamine deficiency	Improved brain delivery and sustained release	Preclinical	[215]
Talineuren (liposomal GM1 formulation)	Neurodegeneration and impaired neuronal homeostasis	Enhanced systemic delivery and bioavailability of GM1 to support neuroprotective and potentially disease-modifying effects	Phase I safety, tolerability, and pharmacokinetic evaluation completed; a randomized, placebo-controlled Phase II pilot study has been registered to assess preliminary short-term efficacy (NCT06431971)	[322,323]
LNPs	SNCA, LRRK2, inflammatory genes	RNA and gene delivery	Preclinical	[244]
PLGA nanoparticles	Neuroprotection and neuroinflammation	Controlled drug delivery	Preclinical	[232]
Exosome-based therapeutics	Neuroimmune signaling and gene regulation	Natural vesicle-mediated delivery	Preclinical	[267]
Cerium oxide nanoparticles	Oxidative stress and mitochondrial dysfunction	Intrinsic antioxidant activity	Preclinical	[324]
Biomimetic nanoparticles	Neuroinflammation	Targeted immune modulation	Preclinical	[325]
α-Synuclein-targeted nanovaccines	Protein aggregation	Enhanced immune-mediated clearance	Experimental	[182]
CRISPR/Cas nanocarriers	Genetic determinants of PD	Genome editing and disease modification	Experimental	[326]
Theranostic nanoplatforms	Diagnosis and therapy	Combined imaging and treatment	Early development	[265]
Multifunctional nanotherapeutics	Multiple PD pathways	Integrated neuroprotection and disease modification	Emerging	[268]

Note: The phase-specific clinical development described above applies to Talineuren, a liposomal formulation of GM1, rather than to the liposomal dopamine formulation cited in Ref. [215]. The Phase I NEON trial evaluated safety, tolerability, and pharmacokinetics, whereas the registered randomized Phase II pilot study is designed to estimate preliminary short-term efficacy.

### 9.6 Future directions in neuroimmune nanotherapeutics

Combining multiple technological and biological advancements is likely to be the key to future advancements in PD therapeutics. The growing knowledge of neuron-immune crosstalk has identified novel therapeutic targets beyond classical dopamine replacement approaches [327].

Next-generation nanoplatforms will be able to target multiple functions, including the mechanisms of neuroinflammation, α-synuclein pathology, mitochondrial dysfunction, oxidative stress, and neural regeneration. Systems that are responsive to stimuli and can be modified to the specific microenvironment of each disease can enhance therapeutic precision and efficacy [328].

The use of artificial intelligence for nanoparticle design, advanced biomaterial development, exosome engineering, genome-editing technologies, and personalized medicine is expected to become increasingly important in the future development of therapeutic interventions. Darwish (2025) reported that further development of this technology will be directed towards integrated platforms for integrated diagnosis, monitoring, and therapy within personalized nanomedicine paradigms (Fig. 12) [329].

The current stage of development of major nanotherapeutic approaches is presented in Table 6. Eventually, close collaboration between neuroscientists, immunologists, materials scientists, clinicians, regulatory agencies, and industry partners will be necessary to make this translation a reality. Duwa et al. (2021) and Yin et al. (2026) showed that these multidisciplinary endeavors can lead to a change in paradigm from fundamental insights into neuron-immune crosstalk to clinically effective nanomedicine-based therapeutics that can substantially impact the treatment of PD [265,290].

## 10 Conclusions

### 10.1 Neuroimmune crosstalk as a central driver of PD

PD is a complex disease characterized by more than just the loss of dopaminergic neurons and the accumulation of α-synuclein. Considerable evidence suggests that PD is a multifactorial neuroimmune disease in which the constant interplay between neurons, microglia, astrocytes, peripheral immune cells, and neurovascular components is critical for the initiation and progression of the disease [330]. The pathogenic cycles of dysregulated neuron-immune crosstalk lead to chronic neuroinflammation, oxidative stress, mitochondrial dysfunction, BBB disruption, and α-synuclein propagation, all of which exacerbate neurodegeneration. The appreciation of these interrelated mechanisms has led to a paradigm shift in our understanding of PD pathogenesis. It has provided many new therapeutic opportunities beyond classical dopamine replacement approaches [193].

### 10.2 Nanoplatforms as multifunctional therapeutic tools

In recent years, nanotechnology has enabled unprecedented possibilities to overcome the biological and pharmacological constraints of current therapeutic approaches for PD. Various nanoplatforms, such as lipid-based nanoparticles, polymeric carriers, inorganic nanomaterials, biomimetic systems, EVs, and hybrid nanocarriers, have been shown to enhance drug delivery to the brain, improve drug specificity, and increase the ability of drugs to cross the BBB. They enable a wide range of therapeutic applications, such as neuroimmune modulation, α-synuclein clearance, gene and mRNA delivery, antioxidant protection, mitochondrial restoration, and targeted regulation of inflammatory signaling pathways. Nanomedicine is thus becoming more than just a PD drug delivery tool, and it is also a potentially broad platform for neuroprotective, disease-modifying, and regenerative approaches to PD [201].

### 10.3 From disease modification to neural repair

One of the themes that arose from this review is the shift towards symptomatic treatment to treatments that can modify the disease course and promote neural recovery. Nanotechnology-based strategies have demonstrated great potential to reduce pathological α-synuclein load, mitigate maladaptive neuroinflammation, restore cellular homeostasis, and preserve vulnerable dopaminergic neurons. Furthermore, combining these nanoplatforms with other therapeutic tools, such as stem cell therapy, neurotrophic factor delivery, biomaterial scaffolds, and gene-editing tools, has the potential to restore nigrostriatal connectivity and neural circuits and repair damaged neural tissue. These multipurpose approaches can eventually give rise to complete therapeutic regimes that can simultaneously tackle several pathological aspects of PD.

### 10.4 Remaining translational challenges

Although there have been significant advances in preclinical studies, several challenges remain in translating these findings into clinical use. Future development depends on a variety of factors, including long-term safety, biodistribution, immunogenicity, large-scale manufacturing, reproducibility, regulatory approval, and cost-effectiveness. Furthermore, the biological diversity of PD calls for better classification of PD, active selection of treatments based on patient biomarkers, and more predictive experimental models of PD. To meet these challenges, collaboration among neuroscientists, immunologists, materials scientists, clinicians, engineers, and regulatory agencies is necessary to ensure that promising laboratory discoveries become safe and effective clinical treatments.

### 10.5 Toward precision neuroimmune nanomedicine

Neuroimmunology, nanotechnology, regenerative medicine, and precision therapeutics represent novel frontiers in PD treatment. Future innovations in the discovery of new biomarkers, the design of nanoplatforms aided by artificial intelligence, new theranostic technologies, gene regulation, and the development of personalized medicine will likely drive the next generation of personalized interventions tailored to individual disease profiles. The combination of mechanistic understanding of the interplay between the nervous and immune systems and intelligent, multifunctional nanotherapeutic systems can open the door to future treatments that could deliver a triple therapeutic benefit of neuroprotection, disease modification, and neural repair. Precision neuroimmune nanomedicine provides a promising framework for shifting PD management from symptomatic to personalized treatments that can slow, stop, and potentially reverse disease progression.

### 10.6 Emerging frontiers in PD nanomedicine

Beyond the established nanoplatform paradigms reviewed above, several emerging technological directions are poised to significantly expand the therapeutic landscape for PD. Nanomaterial-assisted in situ vaccine strategies represent a convergence of nanomedicine and immunotherapy. Nanoparticles engineered to co-deliver conformationally specific α-synuclein antigen fragments with tolerogenic adjuvants (e.g., CpG oligonucleotides, MPLA) to lymph nodes can prime antigen-specific Treg responses and tolerogenic B-cell immunity in situ, potentially suppressing pathological CD4^+^ and CD8^+^ T-cell responses directed against α-synuclein epitopes while preserving overall immune competence. This approach circumvents the poor BBB penetration of conventional immunotherapeutics by exploiting peripheral lymph node priming to establish central immune tolerance. Artificial intelligence (AI)-assisted nanomedicine design accelerates formulation optimization and target identification. Machine learning algorithms trained on large datasets of nanoparticle physicochemical and biological activity have been used to predict LNP organ tropism from lipid composition parameters, identify optimal targeting ligand combinations for BBB transcytosis, and design peptide nanostructures with enhanced α-synuclein disaggregation. Generative AI models are increasingly being applied to de novo nanoparticle design, potentially compressing years of trial-and-error formulation development into weeks of research. To address the translational limitations of conventional preclinical animal models discussed in Section 9.3, midbrain organoids and organ-on-a-chip platforms provide unprecedented human-relevant experimental contexts for evaluating the efficacy and toxicity of nanomedicine. Patient-derived iPSC midbrain organoids harboring LRRK2-G2019S or GBA1 mutations recapitulate α-synuclein aggregation, dopaminergic neuron degeneration, and neuroinflammatory signaling in a human genetic background, thereby enabling evaluation of nanoparticle uptake, intracellular trafficking, and therapeutic responses without the confounding effects of differences in the rodent immune system. BBB-on-a-chip microfluidic devices incorporating human brain endothelial cells, pericytes, and astrocytes under physiological flow conditions allow the quantitative measurement of nanoparticle transcytosis efficiency under disease-relevant inflammatory conditions. Together, these platforms offer a critical bridge between rodent preclinical data and human clinical trials, thereby providing human-relevant solutions to the specific model limitations identified in Section 9.3 while potentially improving the predictive validity of nanomedicine evaluation pipelines.

## CRediT authorship contribution statement

**Shu Zhu:** Conceptualization, Writing – original draft, Writing – review & editing. **Zhongting Wang:** Visualization, Writing – original draft, Writing – review & editing. **Xiaoxi Shi:** Validation, Writing – original draft, Writing – review & editing. **Shanshan Zhong:** Supervision, Writing – original draft, Writing – review & editing. **Ghulam Md Ashraf:** Data curation, Writing – original draft, Writing – review & editing. **Kailei Fu:** Software, Writing – original draft, Writing – review & editing. **Yugang Li:** Conceptualization, Data curation, Writing – original draft, Writing – review & editing.

## Funding

No funding

## Declaration of competing interests

The authors declare that they have no known competing financial interests or personal relationships that could have appeared to influence the work reported in this paper.

## Data Availability

Data will be made available on request.
